# Supplementary material for: Development of Melt-Castable Explosive: Targeted Synthesis of 3,5-Dinitro-4-Methylnitramino-1-Methylpyrazole and Functional Derivatization of Key Intermediates
Source: Molecules. 2025 Jun 28;30(13):2796. doi: 10.3390/molecules30132796 (PMC12251357; doi:10.3390/molecules30132796)
Supplement: Supplementary file 1 [file molecules-30-02796-s001.zip › molecules-3697469-supplementary.pdf]

Supporting Information

Table of content

S1. Compound Overview 1
S2. X-ray diffraction 2
S3. Heat of formation calculation 13
S4. Bomb calorimetry 16
S5. NMR spectroscopy 19
S6. Thermal Stability 20
S7. SSRT (small-scale shock reactivity test) 24
S8. Compatibilities 26
S9. AMES Test 29
S10. Experimental part and general methods 30
S11. References 35

S1. Compound Overview

The syntheses and individual compound numbers are shown in the schemes (**S1**–**S3**) below.

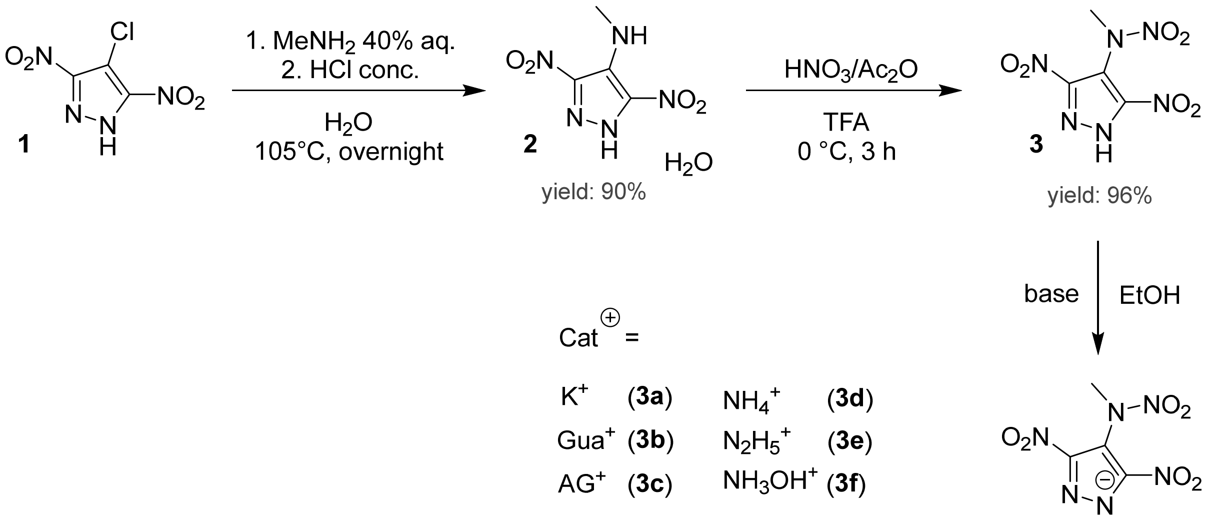

Scheme S1: Synthesis of 3,5-dinitro-4-methylnitraminopyrazole (**3**) and its salts (**3a**–**f**).

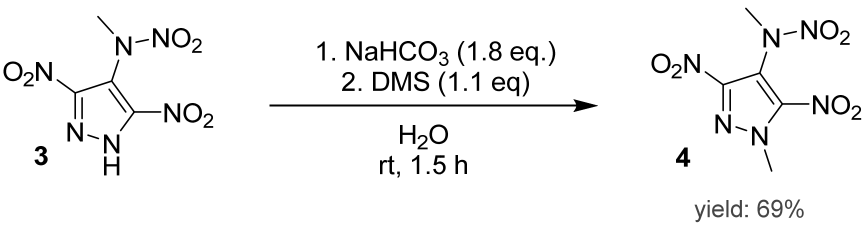

Scheme S2: Synthesis of 3,5-dinitro-4-methylnitramino-1-methylpyrazole (**4**).

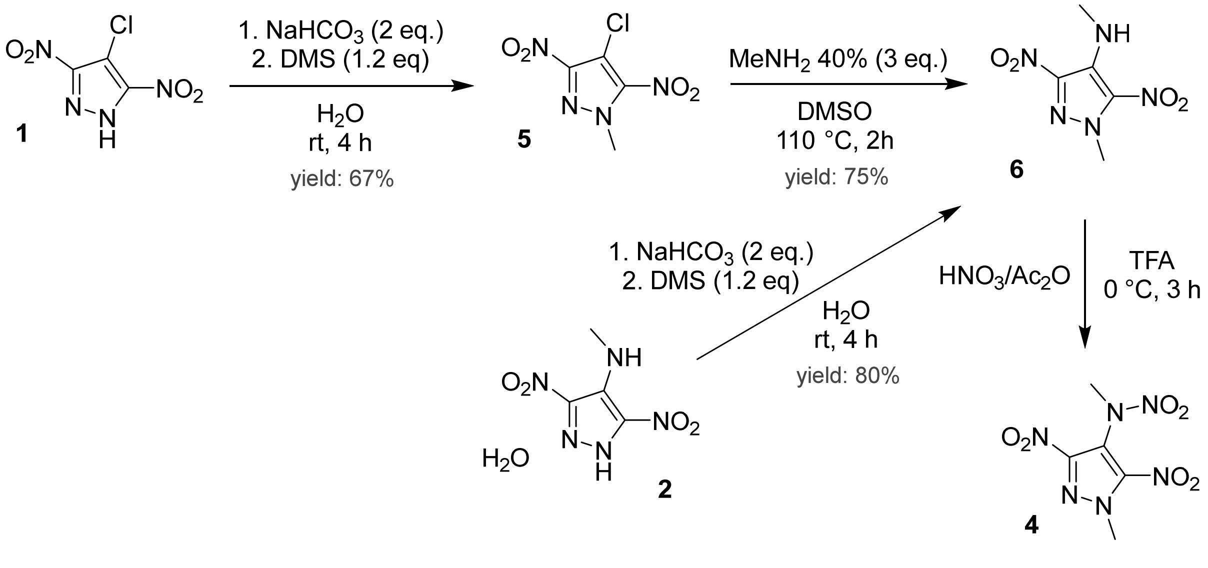

Scheme S3: Alternative synthesis rout of 3,5-dinitro-4-methylnitramino-1-methylpyrazole (**4**).

S2. X-ray diffraction

Crystalline compounds were measured on an *Oxford Xcalibur3* diffractometer with a Spellman generator (voltage 50 kV, current 40 mA) and a Kappa CCD area for data collection using Mo-Kα radiation (λ = 0.71073 Å) or a *Bruker D8 Venture TXS* diffractometer equipped with a multilayer monochromator, a Photon 2 detector and a rotation-anode generator (Mo-Kα radiation). The data collection was performed using the CrysAlisPro software.[S1] The solution of the structure was performed by direct methods and refined by full-matrix least-squares on F2 (SHELXT)[S2] implemented in the OLEX2[S3] software suite. The non-hydrogen atoms were refined anisotropically and the hydrogen atoms were located and freely refined. The absorption correction was carried out by a SCALE3ABSPACK or SADABS Bruker Apex3 multiscan method.[S4,5] All DIAMOND2 plots are shown with thermal ellipsoids at the 50% probability level and hydrogen atoms are shown as small spheres of arbitrary radius.

Table S1: Crystallographic data and structure refinement details for the prepared compounds.

	**DNMNAP (3)**	**K DNMNAP (3a)**	**Gua DNMNAP (3b)**
Formula	$C_4H_4N_6O_6$	$C_4H_3KN_6O_6$	$C_5H_9N_9O_6$
FW [g mol^{-1}]	232.13	270.22	291.21
Crystal system	monoclinic	orthorhombic	monoclinic
Space group	$P2_1$ (No. 4)	*Pbca* (No. 61)	$P2_1/n$ (No. 14)
Color / Habit	colourless/ platelet	yellow/ plate	yellow/ block
Size [mm]	0.03 x 0.08 x 0.10	0.08 x 0.35 x 0.45	0.10 x 0.20 x 0.20
a [Å]	6.0807(2)	12.4865(6)	9.8410(2)
b [Å]	5.8776(2)	9.4728(4)	8.7608(2)
c [Å]	12.1768(5)	15.4906(7)	13.5934(3)
α [°]	90	90	90
β [°]	93.088(1)	90	100.910(2)
γ [°]	90	90	90
V [Å^3]	434.57(3)	1832.26(14)	1150.77(4)
Z	2	8	4
$\rho_{calc.}$ [g cm^{-3}]	1.774	1.959	1.681
μ [mm^{-1}]	0.166	0.616	0.151
F(000)	236	1088	600
$\lambda_{MoK\alpha}$ [Å]	0.71073	0.71073	0.71073
T [K]	109	112	123
θ Min-Max [°]	3.4, 28.4	2.6, 26.4	2.4, 26.4
Dataset	-7:8; -7:7; -16:16	-15:15; -9:11; -16:19	-12:12;-10:10;-16:16
Reflections collected	7670	9342	16870
Independent refl.	2149	1881	2345
R_{int}	0.023	0.032	0.026
Observed reflections	2082	1611	2119
Parameters	151	155	217
R_1 (obs)[a]	0.0309	0.0286	0.0276
wR_2 (all data)[b]	0.0794	0.0687	0.0736
S [c]	1.08	1.03	1.05
Resd. Dens. [e Å^{-3}]	-0.20, 0.28	-0.24, 0.34	-0.22, 0.28
Device type	D8 Venture	Oxford Xcalibur3	Oxford Xcalibur3
Solution	SIR-92	SIR-92	SIR-92
Refinement	SHELXL 2018/1	SHELXL 2018/3	SHELXL 2018/3
Absorption correction	multi-scan	multi-scan	multi-scan
CCDC	2334166	2334159	2334160

[a]$R_1 = \Sigma||F_0|-|F_c||/\Sigma|F_0|$; [b]$wR_2 = [\Sigma[w(F_0^2-F_c^2)^2]/\Sigma[w(F_0)^2]]^{1/2}$; $w = [\sigma c^2(F_0^2)+(xP)^2+yP]^{-1}$ and $P=(F_0^2+2F_c^2)/3$; [c]$S = \{\Sigma[w(F_o^2-F_c^2)^2]/(n-p)\}^{1/2}$ (n = number of reflections; p = total number of parameters).

Table S2: Crystallographic data and structure refinement details for the prepared compounds.

	**AG DNMNAP (3c)**	**NH_4 DNMNAP (3d)**	**Hy DNMNAP (3e)**
Formula	$C_5H_{10}N_{10}O_6$	$C_4H_7N_7O_6$	$C_4H_8N_8O_6$
FW [g mol^{-1}]	306.23	249.17	264.18
Crystal system	monoclinic	orthorhombic	orthorhombic
Space group	$P2_1/n$ (No. 14)	*Pbca* (No. 61)	$P2_12_12_1$ (No. 19)
Color / Habit	yellow/ block	yellow/ plate	colourless/ rod
Size [mm]	0.16 x 0.33 x 0.67	0.05 x 0.20 x 0.20	0.17 x 0.51 x 0.69
a [Å]	12.8059(8)	12.7627(6)	10.6916(7)
b [Å]	6.2685(4)	9.1927(3)	10.8184(6)
c [Å]	15.134(1)	16.3536(6)	17.7605(11)
α [°]	90	90	90
β [°]	92.942(6)	90	90
γ [°]	90	90	90
V [Å^3]	1213.26(13)	1918.66(13)	2054.3(2)
Z	4	8	8
$\rho_{calc.}$ [g cm^{-3}]	1.676	1.725	1.708
μ [mm^{-1}]	0.150	0.160	0.157
F(000)	632	1024	1088
$\lambda_{MoK\alpha}$ [Å]	0.71073	0.71073	0.71073
T [K]	102	122	104
θ Min-Max [°]	2.1, 26.4	2.5, 28.9	2.2, 26.4
Dataset	-16:15; -7:7; -15:18	-17:14; -12:5; -21:19	-13:13;-10:13;-21:22
Reflections collected	9324	10500	14024
Independent refl.	2479	2301	4193
R_{int}	0.025	0.043	0.041
Observed reflections	2059	1701	3477
Parameters	219	182	389
R_1 (obs)[a]	0.0353	0.0377	0.0391
wR_2 (all data)[b]	0.0941	0.0786	0.0758
S [c]	1.03	1.06	1.02
Resd. dens [e Å^{-3}]	-0.30, 0.38	-0.25, 0.32	-0.19, 0.20
Device type	Oxford Xcalibur3	Oxford Xcalibur3	Oxford Xcalibur3
Solution	SHELXL	SIR-92	SIR-92
Refinement	SHELXL 2018/3	SHELXL 2018/3	SHELXL 2018/3
Absorption correction	multi-scan	multi-scan	multi-scan
CCDC	2334157	2334161	2334158

[a]$R_1 = \Sigma||F_0|-|F_c||/\Sigma|F_0|$; [b]$wR_2 = [\Sigma[w(F_0^2-F_c^2)^2]/\Sigma[w(F_0)^2]]^{1/2}$; $w = [\sigma c^2(F_0^2)+(xP)^2+yP]^{-1}$ and $P=(F_0^2+2F_c^2)/3$; [c]$S = \{\Sigma[w(F_o^2-F_c^2)^2]/(n-p)\}^{1/2}$ (n = number of reflections; p = total number of parameters).

Table S3: Crystallographic data and structure refinement details for the prepared compounds.

	**Hx DNMNAP (3f)**	**DNMNAMP (4)**	**CDNMP (5)**
Formula	$C_4H_7N_7O_7$	$C_5H_6N_6O_6$	$C_4H_3ClN_4O_4$
FW [g mol^{-1}]	265.17	246.16	206.55
Crystal system	orthorhombic	monoclinic	monoclinic
Space group	$P2_12_12_1$ (No. 19)	$P2_1/c$ (No. 14)	$P2_1/n$ (No. 14)
Color / Habit	yellow/ block	colourless/ block	colourless/ block
Size [mm]	0.15 x 0.20 x 0.25	0.21 x 0.39 x 1.00	0.50 x 0.50 x 0.60
a [Å]	10.6626(4)	8.2862(7)	5.6620(2)
b [Å]	10.6782(4)	11.6872(8)	8.7290(3)
c [Å]	17.4882(6)	10.5905(9)	15.1705(6)
α [°]	90	90	90
β [°]	90	109.912(10)	97.128(4)
γ [°]	90	90	90
V [Å^3]	1991.16(13)	964.30(15)	743.99(5)
Z	8	4	4
$\rho_{calc.}$ [g cm^{-3}]	1.769	1.696	1.844
μ [mm^{-1}]	0.167	0.155	0.502
F(000)	1088	504	416
$\lambda_{MoK\alpha}$ [Å]	0.71073	0.71073	0.71073
T [K]	123	102	113
θ Min-Max [°]	2.2, 26.4	2.6, 26.4	2.7, 26.4
Dataset	-13:12;-13:12;-20:21	-10:9; -14:14; -13:13	-5:7 ; -10:10 ; -18:18
Reflections collected	16825	9162	7981
Independent refl.	4059	1973	1504
R_{int}	0.033	0.028	0.020
Observed reflections	3674	1683	1406
Parameters	378	178	130
R_1 (obs)[a]	0.0306	0.0333	0.0241
wR_2 (all data)[b]	0.0763	0.0832	0.0609
S [c]	1.04	1.05	1.06
Resd. Dens. [e Å^{-3}]	-0.19, 0.20	-0.19, 0.25	-0.19, 0.26
Device type	Oxford Xcalibur3	Oxford Xcalibur3	Oxford Xcalibur3
Solution	SIR-92	SHELXT	SHELXT
Refinement	SHELXL-2018/3	SHELXL-2018/3	SHELXL-2018/3
Absorption correction	multi-scan	multi-scan	multi-scan
CCDC	2334165	2334164	2334163

[a]$R_1 = \Sigma||F_0|-|F_c||/\Sigma|F_0|$; [b]$wR_2 = [\Sigma[w(F_0^2-F_c^2)^2]/\Sigma[w(F_0)^2]]^{1/2}$; $w = [\sigma c^2(F_0^2)+(xP)^2+yP]^{-1}$ and $P=(F_0^2+2F_c^2)/3$; [c]$S = \{\Sigma[w(F_o^2-F_c^2)^2]/(n-p)\}^{1/2}$ (n = number of reflections; p = total number of parameters).

Table S4: Crystallographic data and structure refinement details for the prepared compounds.

	**DNMAMP (6)**
Formula	$C_5H_7N_5O_4$
FW [g mol^{-1}]	201.16
Crystal system	orthorhombic
Space group	*Pbca* (No. 61)
Color / Habit	orange/ block
Size [mm]	0.35 x 0.50 x 0.50
a [Å]	10.9652(4)
b [Å]	13.3029(3)
c [Å]	21.8848(7)
α [°]	90
β [°]	90
γ [°]	90
V [Å^3]	3192.31(17)
Z	16
$\rho_{calc.}$ [g cm^{-3}]	1.674
μ [mm^{-1}]	0.145
F(000)	1664
$\lambda_{MoK\alpha}$ [Å]	0.71073
T [K]	113
θ Min-Max [°]	2.6, 26.4
Dataset	-13:7; -16:16; -27:26
Reflections collected	24473
Independent refl.	3261
R_{int}	0.058
Observed reflections	2505
Parameters	309
R_1 (obs)[a]	0.0386
wR_2 (all data)[b]	0.0982
S [c]	1.05
Resd. Dens. [e Å^{-3}]	-0.22, 0.23
Device type	Oxford Xcalibur3
Solution	SHELXT
Refinement	SHELXL-2018/3
Absorption correction	multi-scan
CCDC	2334162

[a] $R_1 = \Sigma||F_0|-|F_c||/\Sigma|F_0|$; [b] $wR_2 = [\Sigma[w(F_0^2-F_c^2)^2]/\Sigma[w(F_0)^2]]^{1/2}$; $w = [\sigma c^2(F_0^2)+(xP)^2+yP]^{-1}$ and $P=(F_0^2+2F_c^2)/3$; [c] $S = \{\Sigma[w(F_o^2-F_c^2)^2]/(n-p)\}^{1/2}$ (n = number of reflections; p = total number of parameters).

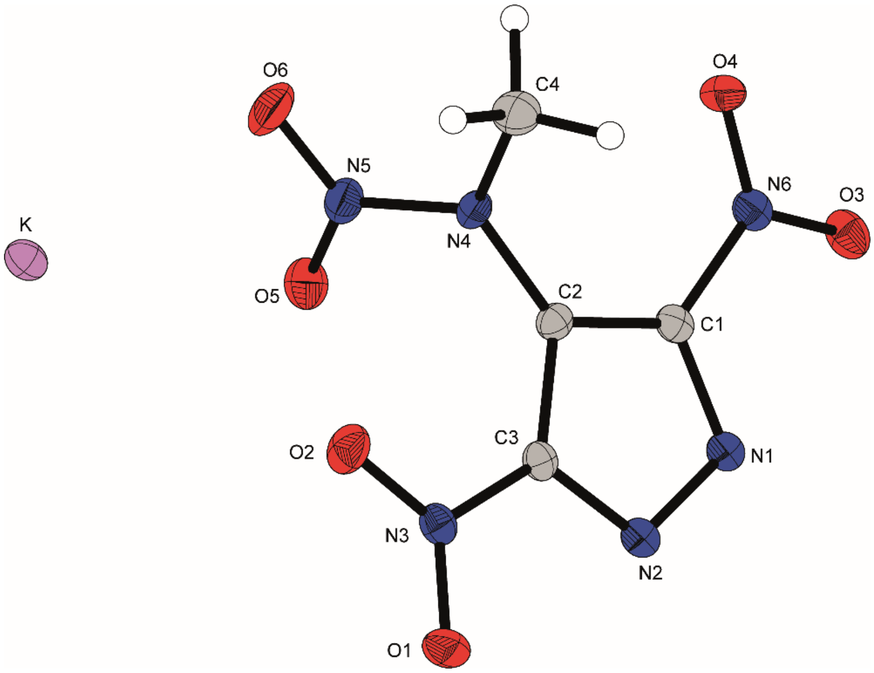

Figure S1: X-ray structure of potassium salt of compound **3** (**3a**).

Potassium salt of compound **3** crystallizes from ethanol in form of yellow block in the orthorhombic space group *P*bca with eight molecules per unit cell, a cell volume of 1832.26(10) $Å^3$ and a calculated density of 1.959 g cm^{-3} at 112 K. The molecular unit is illustrated in **Figure S1**. Potassium atom is coordinated irregularly the nitrogen atom n1, N2 and the oxygen atoms O1, O3, O4, O5 and O6. Thereby, oxygen O3 takes a special role by the bridging two potassium atoms, whereas other oxygen and nitrogen atoms only coordinate with one potassium atom. The length of K−N1 and K−N2 are 2.859 and 2.834 Å respectively and are only minimal shorter than the average length between the potassium atom and the oxygen atoms of 2.862 Å. One 3,5-dinitro-4-methylnitraminopyrazolat anion forms eight coordination bonds with the surrounding potassium ions.

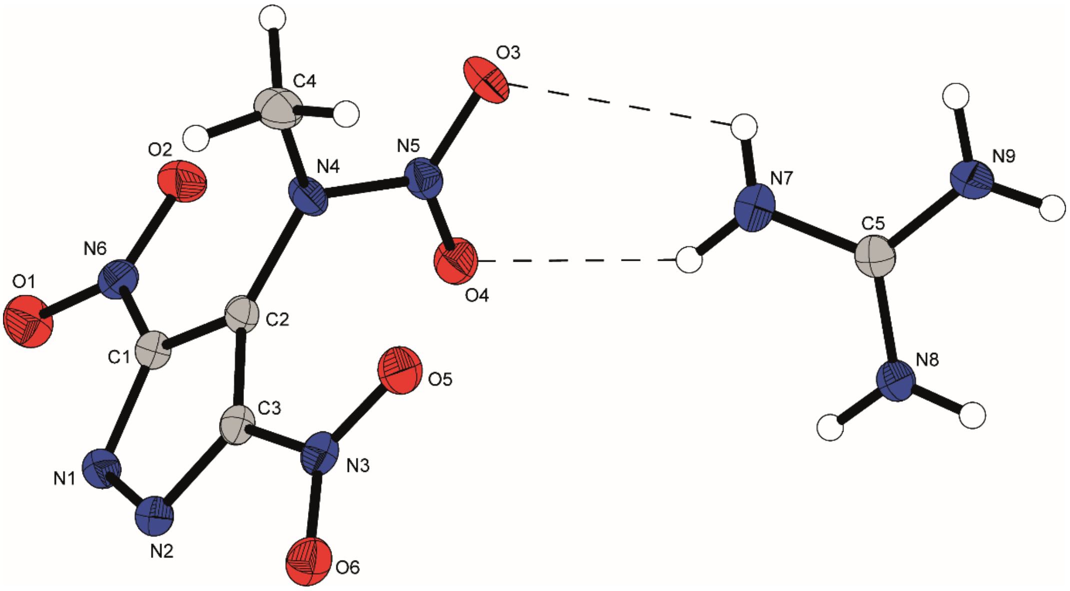

Figure S2: X-ray structure of guanidinium salt of compound **3** (**3b**).

Guanidinium salt of compound **3** crystallizes from water in form of yellow block in the monoclinic space group $P2_1/c$ with four molecules per unit cell, a cell volume of 1150.77(4) Å^3 and a calculated density of 1.681 g cm^{-3} at 123 K. The molecular unit is illustrated in **Figure S2**. The guanidinium cation is coordinated regularly 3,5-dinitro-4-methylnitraminopyrazolate anions via hydrogen bonding. As illustrated in **Figure S2**, the hydrogen atoms of nitrogen N7 coordinate to nitro oxygen O3 and O4 of the anion. Furthermore, nitrogen atom N1 and N2 interoperate with N8H and N9H, respectively, whereas O5 and O6 coordinate to the remaining two hydrogen atoms of nitrogen N8 and N9, respectively. The methyl group forms non-classic hydrogen bonds to the oxygens O3 and O6.

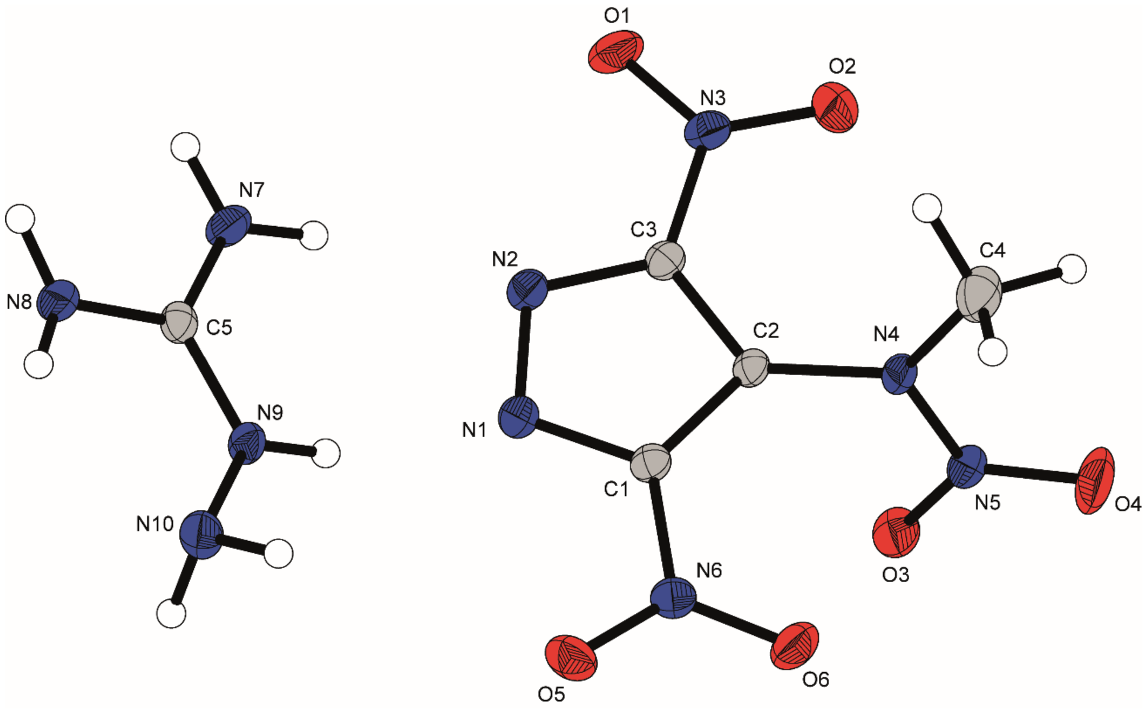

Figure S3: X-ray structure of aminoguanidinium salt of compound **3** (**3c**).

Aminoguanidinium salt of compound **3** crystallizes from ethanol in form of yellow block in the monoclinic space group $P2_1/n$ with four molecules per unit cell, a cell volume of 1213.26(13) Å^3 and a calculated density of 1.676 g cm^{-3} at 102 K. The molecular unit is illustrated in **Figure S3**. Similar to guanidinium, the aminoguanidinium cation coordinates via its N7H and N9H protons with the N2 and N3 nitrogens of the pyrazole through hydrogen bonding. However, no association with the nitro group of the methylnitramine is observed, as in compound **3b**. It can be observed that the pyrazol anion and aminoguanidinium cation do not lie in the same plane. The plane of the aminoguanidinium cation is offset by 30.52(7) °.

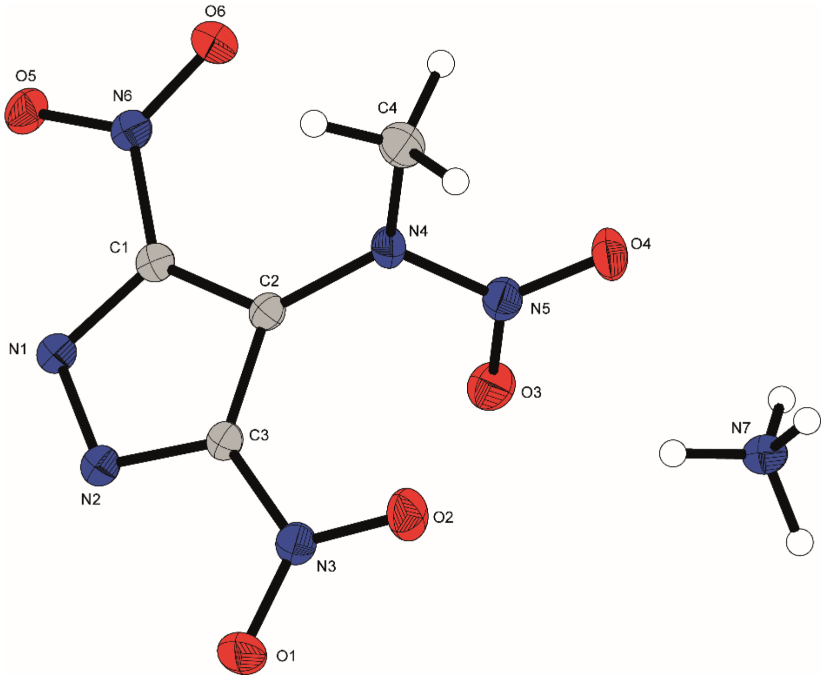

Figure S4: X-ray structure of ammonium salt of compound **3** (**3d**).

Ammonium salt of compound **3** crystallizes from water in form of yellow plate in the orthorhombic space group *Pbca* with eight molecules per unit cell, a cell volume of 1918.66(13) $Å^3$ and a calculated density of 1.725 g cm^{-3} at 122 K. Molecular unit is illustrated in **Figure S4**. The ammonium cation is coordinated via hydrogen bonding by the nitrogen atoms N1, N2 and the oxygen atoms O3 and O5. The lengths of N7H···N1 and N7H···N2 are 2.009 and 1.999 Å, respectively, which are only minimal shorter on average than the lengths of N7H···O3 (2.039 Å) and N7H···O5 (2.102 Å). Obviously, one 3,5-dinitro-4-methylnitraminopyrazolate anion forms four coordination hydrogen bonds with the surrounding ammonium cations.

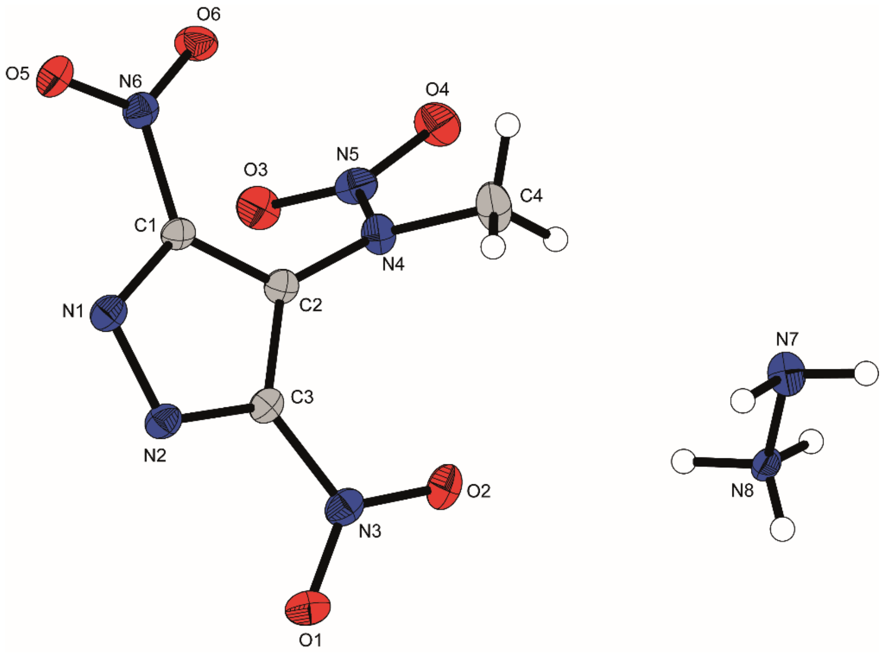

Figure S5: X-ray structure of hydrazinium salt of compound **3** (**3e**).

Hydrazinium salt of compound **3** crystallizes from ethanol in form of colourless rod in the orthorhombic space group $P2_12_12_1$ with eight molecules per unit cell, a cell volume of 2054.3(2) Å^3 and a calculated density of 1.708 g cm^{-3} at 104 K. The molecular unit is illustrated in **Figure S5**. Pyrazolate anion is coordinating via hydrogen bond by nitrogen atoms N1 and N2 to the protons H7 and H8^i of two hydrazinium cation, respectively. Each of the hydrazinium cations is coordinating to another hydrazinium or pyrazolate. The length of connection between two hydrazinium ions is with 2.176 Å longer as the hydrogen bonds between hydrozinium and pyrazolate ions (N1···H7A 2.080 Å, N2···H8D 1.919 Å).

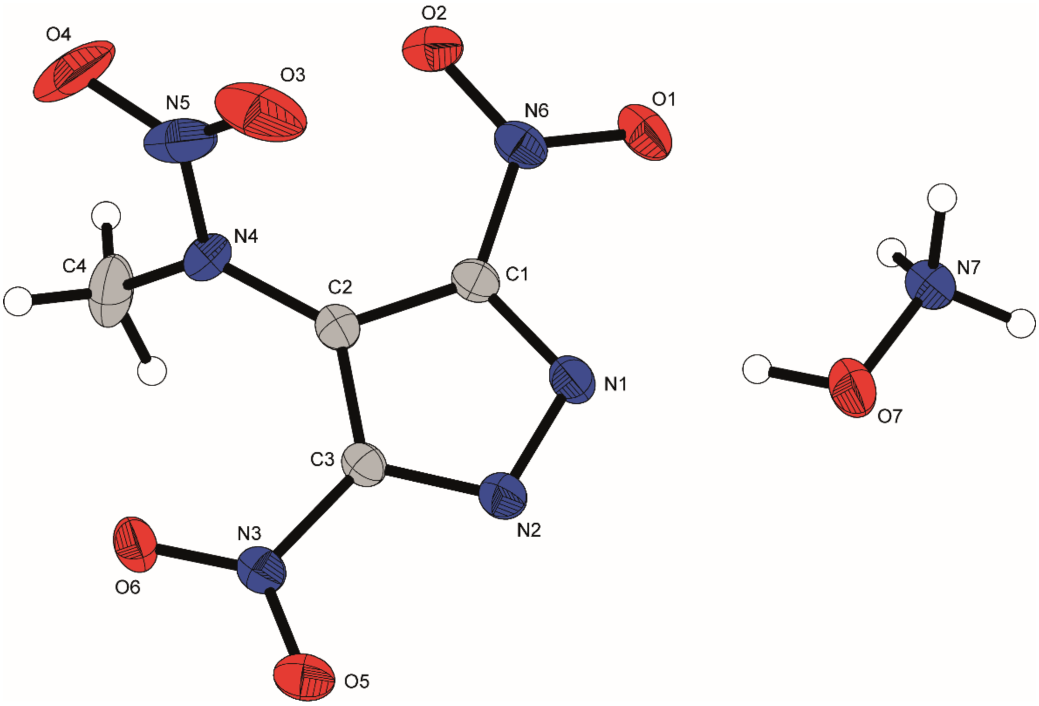

Figure S6: X-ray structure of hydroxylammonium salt compound **3** (**3f**).

Hydroxylammonium salt of compound **3** crystallizes from water in form of yellow block in the orthorhombic space group $P2_12_12_1$ with eight molecules per unit cell, a cell volume of 451.6(3) Å^3 and a calculated density of 1.612 g cm^{-3} at 173 K. The molecular unit is illustrated in **Figure S6**. The packing is strongly influenced by the different hydrogen bonds that are all build up on the hydroxylammonium cation. **Figure S6** illustrates the hydrogen-bonding between the hydrogen atom of oxygen O7 and the adjacent nitrogen atom N1 of the pyrazole. Moreover, the hydrogen atom N7H coordinats to the nitrogen atom N2^i of the second pyrazole anion. Each pyrazole molecule forms hydrogen bonds with four hydroxylammonium cations, and conversely, each cation interacts with four pyrazole molecules. The coordination distances rang from 1.744 to 2.481 Å, where the shortest length is represented by the previously mentioned O7H···N1-hydrogen bonding while the losnges coordination was observed between hydrogen atom N7H and oxygen atom O5.

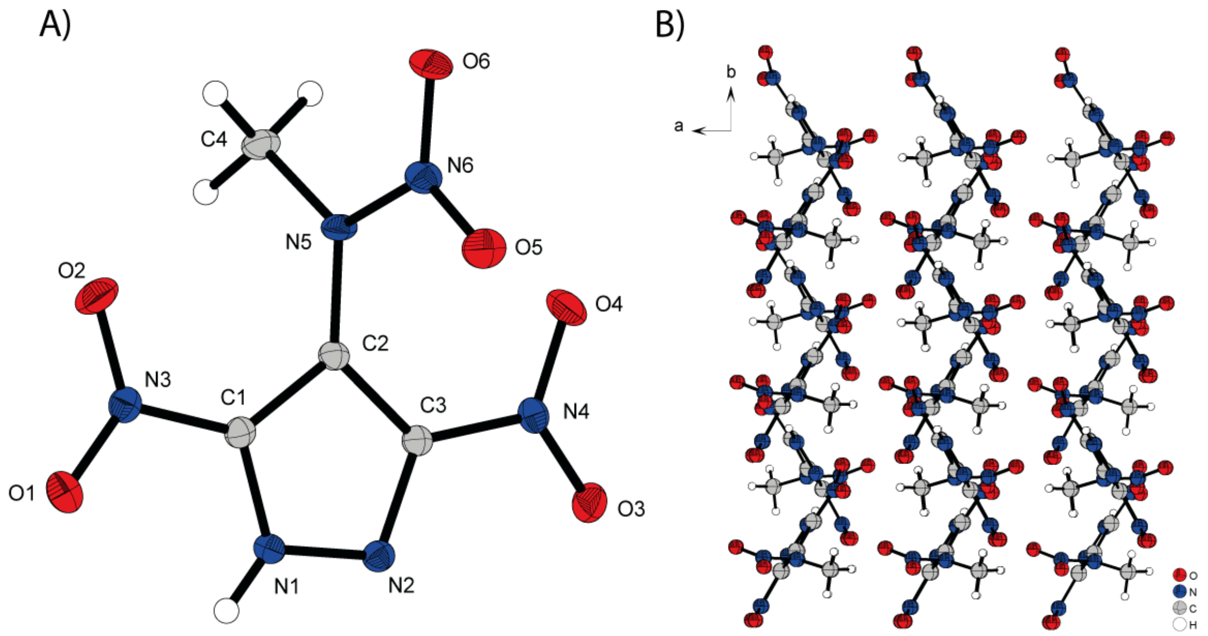

Figure S7: X-ray structure of compound **3**.

3,5-Dinitro-4-methylnitraminopyrazole **(3)** crystallizes from ethanol in form of colourless plates in the monoclinic space group $P2_1$ with four molecules per unit cell, a cell volume of 434.57(3) Å^3 and a calculated density of 1.774 g cm^{-3} at 109 K. The molecular unit is illustrated in **Figure S7A** and the extended structure is illustrated in **Figure S7B**.

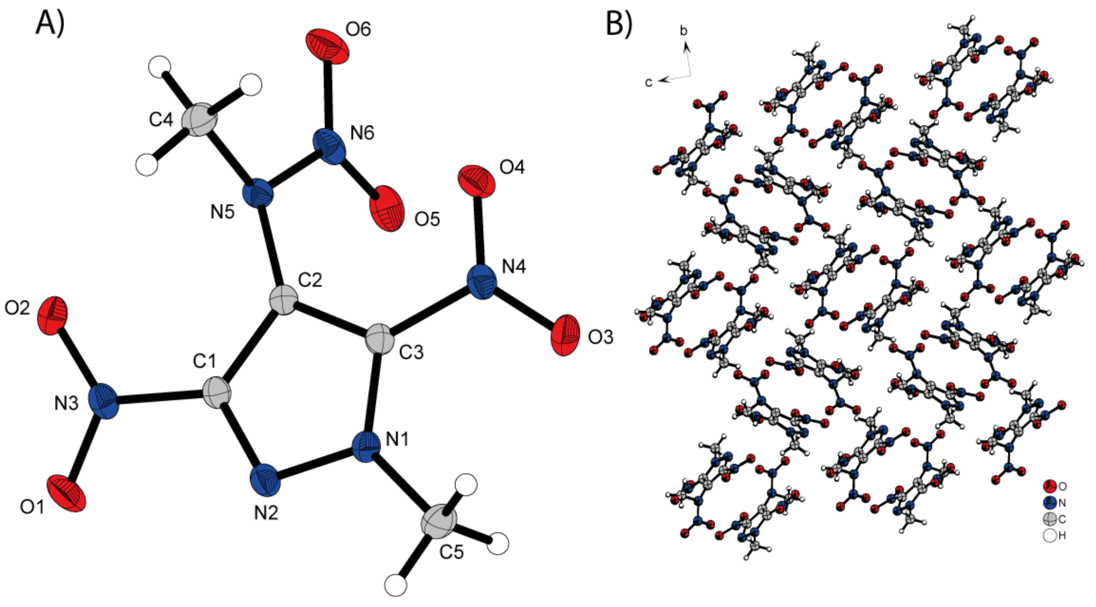

Figure S8: X-ray structure of compound **4**.

Compound **4** crystallizes from acetonitrile in form of colourless blocks in the monoclinic space group $P2_1/c$ with four molecules per unit cell, a cell volume of 964.30(14) Å^3 and a calculated density of 1.696 g cm^{-3} at 102 K. The molecular unit is illustrated in **Figure S8A** and the extended structure is illustrated in **Figure S8B**.

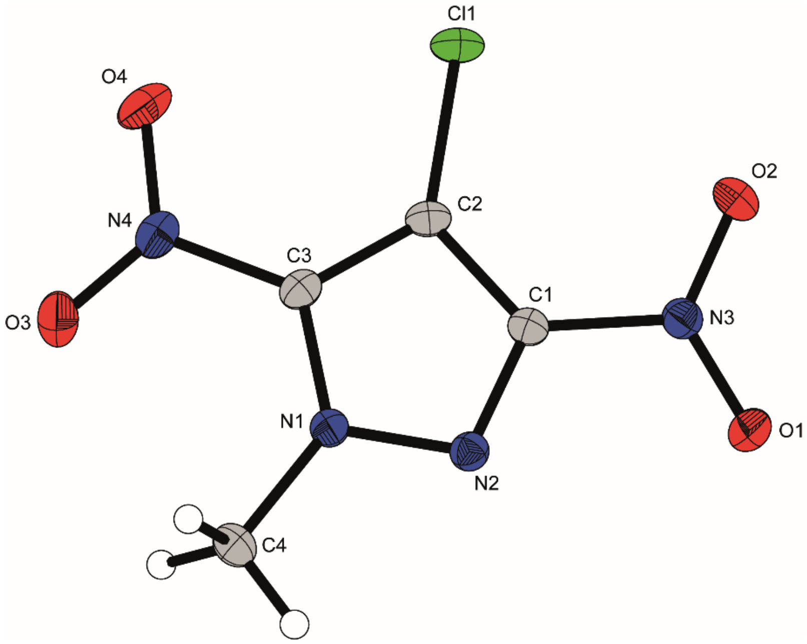

Figure S9: X-ray structure of compound **5**.

3,5-Dinitro-4-methylamino-1-methylpyrazole **(5)** crystallizes from ethanol in form of colourless block in the monoclinic space group $P2_1/n$ with four molecules per unit cell, a cell volume of 743.99(5) Å^3 and a calculated density of 1.844 g cm^{-3} at 113 K. The molecular unit is illustrated in **Figure S9**.

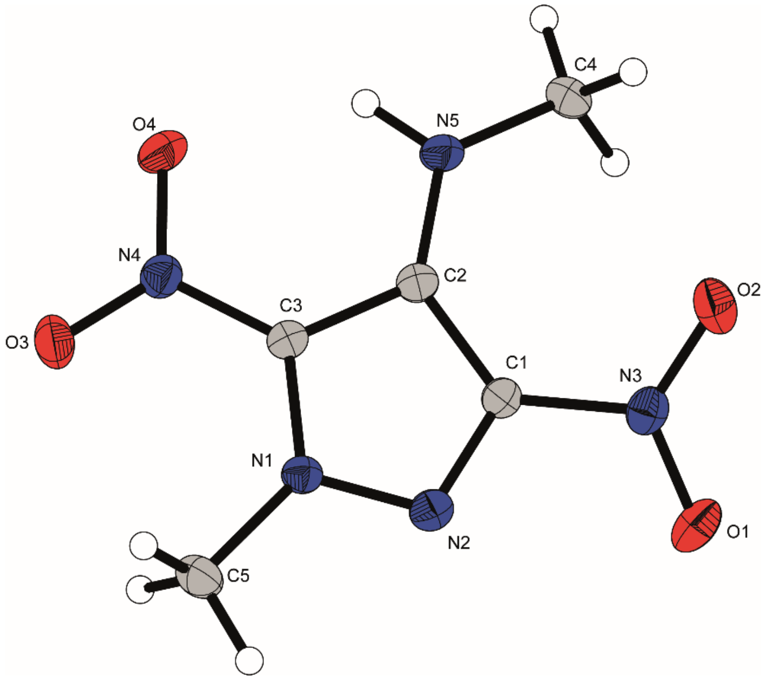

Figure S10: X-ray structure of compound **6**.

Compound **6** crystallizes from methanol in form of orange blocks in the orthorombic space group *Pbca* with sixteen molecules per unit cell, a cell volume of 3192.31(17) Å^3 and a calculated density of 1.674 g cm^{-3} at 113 K. The molecular unit is illustrated in **Figure S10**.

S3. Heat of formation calculation

All quantum chemical calculations were performed using the Gaussian G09 program package.[S6] The complete basis set (CBS) method of Petersson and coworkers was used for calculation of enthalpies (H) and free energies (G), listed in Table S6 in order to obtain very accurate energies. The CBS models use the known asymptotic convergence of pair natural orbital expressions to extrapolate from calculations using a finite basis set to the estimated complete basis set limit. CBS-4 starts with a HF/3-21G(d) geometry optimization; the zero-point energy is computed at the same level. It then uses a large basis set SCF calculation as a base energy, and a MP2/6-31+G calculation with a CBS extrapolation to correct the energy through second order. A MP4(SDQ)/6-31+(d,p) calculation is used to approximate higher order contributions. In this study, we applied the modified CBS-4M method (M referring to the use of minimal population localization), which is a re-parametrized version of the original CBS-4 method and also includes some additional empirical corrections.[S7] The gas-phase enthalpies ($\Delta_f H°(g, M, 298)$) of the species were computed according to the atomization energy method (**Equation S1**) using room temperatures CBS-4M enthalpies (**Table S5**).[S8]

$$\Delta_f H°_{(g, M, 298)} = H_{(molecule, 298)} - \sum H°_{(atoms, 298)} + \sum \Delta_f H°_{(atoms, 298)} \quad \textbf{(Eq. S1)}$$

Table S5: CBS-4M electronic enthalpies for atoms C, H, N and O and their literature values.

	$-H^{298}$ / a.u.	$\Delta_f H°_{gas}$ [S9]
H	0.500991	217.998
C	37.786156	716.68
N	54.522462	472.68
O	74.991202	249.18

In order to obtain the energy of formation for the solid phase of all compounds, the Trouton's Rule has to be applied ($\Delta H_{sub} = 188\ T_m$).[S10]

Table S6: CBS-4M results and calculated gas-phase enthalpies.

	$-H^{298}$ [a] [a.u.]	$\Delta_f H°(g, M)$ [b] [kJ mol^{-1}]
**3 Anion**	−932.738126	−47.1
**3a (K^+)**	−599.187712	89.0
**3b (G^+)**	−205.453192	571.2
**3c (AG^+)**	−260.701802	670.7
**3d (NH_4^+)**	−56.796608	635.3
**3e (Hy^+)**	−112.030523	773.4
**3f (Hx^+)**	−131.863229	686.5
**3**	−933.218152	226.0
**4**	−972.454902	200.8
**6**	−768.182618	152.5

[a] CBS-4M electronic enthalpy; [b] gas phase enthalpy of formation.

These molar standard enthalpies of formation (ΔH_m) were used to calculate the molar solid state energies of formation (ΔU_m) according to Equation **S2** (**Table S7**).

$$\Delta U_m = \Delta H_m - \Delta n\, RT \quad \textbf{(Eq. S2)}$$

Table S7: Heat of formation calculation results for compounds **3**–**4** and **6**.

	V_M[a] [nm^3]	$\Delta_f H°(s)$[b] [kJ mol^{-1}]	Δn[c]	M [g mol^{-1}]	$\Delta_f U(s)$ [d] [kJ kg^{-1}]
**3a**	0.229	−446.5	−7.5	270.20	−1583.8
**3b**	0.288	60.0	−12.0	291.18	308.2
**3c**	0.303	165.7	−13.0	306.20	646.5
**3d**	0.240	101.9	−10.0	249.14	508.5
**3e**	0.257	248.5	−11.0	264.16	1044.0
**3f**	0.249	157.7	−10.5	265.14	693.0
**3**	/	145.1	−8.0	232.11	710.7
**4**	/	135.0	−9.0	246.14	639.1
**6**	/	76.1	−8.0	201.14	477.2

[a] Molecular volumes taken from X-ray structure and corrected to room temperatures; [b] standard solid state enthalpy of formation; [c] the change of moles of gaseous components when formed; [d] solid state energy of formation.

Tables **S8** and **S9** summarize the physiochemical properties and detonation parameters of the salts of compound **3** (**3a**, **3b**, **3c**, **3d**, **3e**, **3f**) and neutral compounds **3**, **4** and **6**.

Table S8: Physicochemical properties and detonation parameter of salts of compound **3** (**a**, **b**, **c**, **d**, **e**, **f**) compared to **TNT**.

	**(3a)**	**(3b)**	**(3c)**	**(3d)**	**(3e)**	**(3f)**	**TNT**[n]
Formula	$C_4H_3KN_6O_6$	$C_5H_9N_9O_6$	$C_5H_{10}N_{10}O_6$	$C_4H_7N_7O_6$	$C_4H_8N_8O_6$	$C_4H_7N_7O_7$	**$C_7H_5N_3O_6$**
FW [g·mol^{-1}]	270.20	291.18	306.20	249.14	264.16	265.14	**227.13**
IS[a] [J]	6	>40	8	3	5	5	**15**
FS[b] [N]	80	>360	168	192	120	192	**>360**
ESD[c] [mJ]	140	1.2	750	600	480	370	
Ω_{CO2}[d] [%]	−24	−47	−47	−35	−36	−27	**−74.0**
T_{endo}[e] [°C]	/	140	116	/	/	/	**81**
$T_{exo.}$[f] [°C]	217	158	148	169	165	147	**289**
ρ[g] [g·cm^{-3}]	1.906	1.638	1.628	1.681	1.660	1.724	**1.65**
$\Delta_f H°$[h] [kJ·mol^{-1}]	-446.5	60.0	165.7	101.9	248.5	157.7	**−185**
**EXPLO5 V6.05 values**							
$-\Delta_E U°$[i] [kJ·kg^{-1}]	3676	4414	4612	5205	5530	5798	**5022**
T_{C-J} [j] [K]	2810	3104	3177	3545	3674	3860	**3452**
p_{C-J} [k][GPa]	21.7	22.7	23.3	26.8	27.3	30.5	**20.5**
D_{C-J} [l] [m·s^{-1}]	7296	7719	7848	8129	8261	8470	**6950**
V^0 [m] [dm^3·kg^{-1}]	540	814	834	798	825	786	**633**

[a] Impact sensitivity (BAM drophammer, method 1 of 6); [b] friction sensitivity (BAM friction tester, method 1 of 6); [c] electrostatic discharge device (OZM research, method 1 of 6); [d] oxygen balance toward carbon dioxide ($\Omega_{CO2} = (n\text{O} - 2x\text{C} - y\text{H}/2)(1600/\text{FW})$); [e] endothermic event (DTA, β = 5 °C·min^{-1}); [f] temperature of decomposition (DTA, β = 5 °C·min^{-1}); [g] density at 298 K recalculated from X-ray data; [h] heat of formation (calculated using the atomization method and CBS-4M enthalpies; [i] detonation energy; [j] detonation temperature; [k] detonation velocity; [l] detonation pressure; [m] volume of detonation gases at standard temperature and pressure conditions; [n] determined at LMU.

Table S9: Physicochemical properties and detonation parameter of compounds **6**, **3** and **4** compared to **TNT**.

	**(6)**	**(3)**	**(4)**	**TNT**[n]
Formula	$C_5H_7N_5O_4$	$C_4H_4N_6O_6$	$C_5H_6N_6O_6$	**$C_7H_5N_3O_6$**
FW [g·mol−1]	201.14	232.11	246.14	**227.13**
IS[a] [J]	>40	8	15	**15**
FS[b] [N]	>360	144	>360	**>360**
ESD[c] [mJ]	/		740	
Ω_{CO2}[d] [%]	−75.6	−27.6	−45	**−74.0**
T_{endo}[e] [°C]	133	/	77	**81**
$T_{exo.}$[f] [°C]	203	157	190	**289**
ρ[g] [g·cm−3]	1.629	1.725	1.648	**1.65**
$\Delta_f H°$[h] [kJ·mol−1]	76.1	145.1	135.2/106.4[o]	**−185**
**EXPLO5 V6.05 values**				
$-\Delta_E U°$[i] [kJ·kg−1]	4316	5457	5147/5040[o]	**5022**
T_{C-J} [j] [K]	2934	3924	3603/3547[o]	**3452**
p_{C-J} [k][GPa]	19.6	28.8	24.1/23.8[o]	**20.5**
D_{C-J} [l] [m·s−1]	7276	8228	7721/7682[o]	**6950**
V^0 [m] [dm3·kg-1]	760	743	752/752[o]	**633**

[a] Impact sensitivity (BAM drophammer, method 1 of 6); [b] friction sensitivity (BAM friction tester, method 1 of 6); [c] electrostatic discharge device (OZM research, method 1 of 6); [d] oxygen balance toward carbon dioxide ($\Omega_{CO2} = (nO - 2xC - yH/2)(1600/FW)$); [e] endothermic event (DTA, β = 5 °C·min−1); [f] temperature of decomposition (DTA, β = 5 °C·min−1); [g] density at 298 K recalculated from X-ray data; [h] heat of formation (calculated using the atomization method and CBS-4M enthalpies; [i] detonation energy; [j] detonation temperature; [k] detonation velocity; [l] detonation pressure; [m] volume of detonation gases at standard temperature and pressure conditions; [n] determined at LMU; [o] value from bomb calorimeter.

S4. Bomb calorimetry

The bomb calorimetry experiments were performed using a Parr isoperibol oxygen bomb calorimeter, Model 6200, equipped with a *Parr* 1104 High Strength Vessel with Cage and Loop is a heavy-walled, 240 mL vessel, designed for combustion tests of explosive materials. Additionally, a platinum crucible, fuse wire (Ni-Cr alloy) and ignition thread (cotton) were employed. Benzoic acid was employed as a burning aid during the measurements, and the bomb's calibration was performed using benzoic acid (*Parr*, one gram pellets, standardized for bomb calorimetry) as a reference standard. The energy of combustion of the benzoic acid is $\Delta_c U°$ = 26414 J g^{-1}, and the energy equivalent of the used bomb is ε = 10570.52 J K^{-1}. In the measurement process, both the test substance and benzoic acid were accurately weighed (in grams, measured to four decimal places) and thoroughly homogenized by grinding. The resulting fine powder was then compressed into tablets using a hydraulic press exerting 3 tons of pressure with a diameter of 12.9 mm. The tablets were then precisely weighed (in grams with four decimal places). The weight percentage of benzoic acid was recorded as the burning aid, while the weight percentage of the substance being analyzed was registered as the sample weight. The proportions were meticulously chosen to ensure a minimum temperature increase of 2 K in the water during the measurement phase. Subsequently, 1 mL of distilled water was introduced into the bomb, and the system was pressurized with 30 bar of oxygen. Post-measurement, the bomb was cleansed using distilled water. Its acidity was assessed by titration using a 0.1 M NaOH solution (*Fisher Chemicals*), and the endpoint indicated using bromothymol blue (*Merck*) as an indicator. Each investigated substance underwent a series of three measurements, from which the mean value was calculated.

When analyzing substances with a high percentage nitrogen content, it is recommended to implement an acid correction. This is because the generation of nitric acid during the combustion process can slightly influences the measurement of the combustion heat. This influence can be quantified by titration. This quantification allows for a precise adjustment in the calculations of the energy balance, ensuring that the measurements of combustion heat are accurate and account for any changes caused by acid formation. The acid correction can be carried out according to equation **S3**.

$$\Delta U_{corr} = \Delta U - (\Delta_f H°_{HNO3} * V_{NaOH} * c_{NaOH}) \qquad \textbf{(Eq. S3)}$$

Here ΔU is the energy of combustion (cal/g), $\Delta_f H°_{HNO3}$ is the enthalpy of formation of nitric acid (−57.7 kJ/mol)[S11], c_{NaOH} is the concentration of NaOH and V_{NaOH} is the volume of NaOH used in the titration. All values can be find in Table **S10**.

Table S10: Report of the experimental data from bomb calorimeter from the Combustion Calorimeter Tool – Experiment Mode (NIST)[S12].

**DNMNAMP (4)**: $\Delta_c U° = -11927.41\ \mathrm{J \cdot g^{-1}}\ (-2850.72\ \mathrm{cal \cdot g^{-1}})$			
m(3,4-DNPz)/ g	0.480402	0.489547	0.539836
m'(benzoic acide)/ g	0.559105	0.559305	0.562668
m''(NiCr)/ g	0.015900	0.015900	0.016700
m'''(Cotton)/ g	0.010407	0.010507	0.010807
m(soot)/ mg	0.00	0.00	0.00
$p(O_2)$/ MPa	3.00	3.00	3.00
T_i/ K	297.45150	297.54840	297.67410
T_f/ K	299.42370	299.53160	299.72360
ΔT_c/ K	1.97220	1.98320	2.04950
ε^i/ $J \cdot K^{-1}$	11.87	11.87	11.87
ε^f/ $J \cdot K^{-1}$	13.06	13.06	13.14
Δm_{H2O}/ (g)	0.00	0.00	0.00
$\Delta_{ign}u$/ J	20	20	20
$-m' \cdot \Delta_c u'$/ J	14768.20	14773.49	14862.31
$-m'' \cdot \Delta_c u''$/ J	93.12	93.12	97.81
$-m''' \cdot \Delta_c u'''$/ J	176.34	178.04	183.12
$\varepsilon \cdot (-\Delta T_c)$/ J	−20847.20	−20963.47	−21664.30
$\varepsilon_{cont} \cdot (-\Delta T_c)$/ J	−24.92	−25.19	−26.33
$\Delta_{IBP}u$/ J	−20852.11	−20968.66	−21670.63
m(soot)$\cdot \Delta_c u_{soot}$/ J	0.00	0.00	0.00
$\Delta u(HNO_3)$/ J	63.88	66.27	69.85
Δu_{corr}/ J	18.99	19.14	20.06
$\Delta_c u°$(cr)/ $J \cdot g^{-1}$	−11930.79	−11926.55	−11924.89

m is the mass of the sample of the investigated compound adjusted to vacuum conditions; m', m'', and m''' are the masses of the auxiliary substances adjusted to vacuum conditions; m(soot) is the mass of the soot formed in the experiment; $p(O_2)$ is the initial pressure of oxygen in the bomb; T_i and T_f are the initial and final temperatures in the main period; ΔT_c is the corrected temperature rise; ε^i and ε^f are the heat capacity of the contents of the bomb in the initial and final states, respectively; $\Delta_c u'$, $\Delta_c u''$, and $\Delta_c u'''$ are the specific combustion energies of the auxiliary compounds; $\Delta_{ign}u$ is the energy for igniting the sample; ε is the energy equivalent of the calorimeter; $\varepsilon_{cont}(-\Delta T_c) = \varepsilon^i (T_i - 298.15) + \varepsilon^f (298.15 - T_f + \Delta T_{corr})$, where ΔT_{corr} is the temperature correction for the heat of stirring and the heat exchange between the calorimeter and its environment; + $\Delta_c u_{soot}$ is the average combustion energy of the soot (-32.8 kJ g^{-1}); Δ_{IBP}u is the change of internal energy for the isothermal bomb process; $\Delta u(HNO_3)$ is the energy required for decomposition of the HNO_3 solution formed into N_2, O_2, and H_2O(l); Δu_{corr} is the energy correction to the standard state. For the sulfur compounds, the final result refers to the H_2SO_4*115H_2O solution. Δm_{H2O}, set to 0 for most cases, is for correcting the mass of water outside the bomb.

The conversion of combustion energy $\Delta_c U$ from calories per gram (cal/g) to kilojoules per gram (kJ/g) is accomplished through the relationship 1 cal/g = 4.184 J/g.

The combustion energy $\Delta_c U$ acquired in kilojoules per gram (kJ/g) can be transformed into the enthalpy of combustion $\Delta_c H$ according to Equation **S4**.

$$\Delta_c H = (\Delta U * M) + \Delta n_g RT \qquad \textbf{(Eq. S4)}$$

Where R is the ideal gas constant, T is the temperature at which the combustion takes place; M is the molar mass and Δng represents the molar change of the gases participating in the combustion process. To calculate Δn_g, knowledge of the specific combustion reaction equation is required.
The reaction equation for combustion process is expressed as:

$$C_aH_bO_cN_d + \left(a + \frac{b}{4} - \frac{c}{2}\right) O_{2(g)} \rightarrow aCO_{2(g)} + \frac{b}{2} H_2O_{(l)} + \frac{d}{2} N_{2(g)}$$

Based on this equation, the expression for Δn_g is determined as:

$$\Delta n_g = aCO_{2(g)} + \frac{d}{2} N_{2(g)} - \left(a + \frac{b}{4} - \frac{c}{2}\right) O_{2(g)} = \frac{1}{2}\left(d - \frac{b}{2} + c\right)$$

This relation provides the change in the number of moles of gases involved in the combustion, taking into account the specific reaction and assumptions about the oxidation states of hydrogen, carbon, and the release of nitrogen as N_2[S11].

The determination of the enthalpy of formation $\Delta_f H$ based on the enthalpy of combustion $\Delta_c H$ can be achieved through the application of thermodynamic principles. The reformulated expression is given by Equation **S7**.

$$\Delta_f H°_{reactants} = \sum \Delta_f H°_{products} - \Delta_c H° \qquad \textbf{(Eq. S7)}$$

The enthalpies of formation for the reaction products were sourced from the National Institute of Standards and Technology (NIST) database (Table **S11**)[S9].

Table S11: Enthalpies of formation of the reaction products.

Compound	$\Delta_f H°$ [kJ/mol]
CO_2	−393.52
H_2O	−285.83
N_2	0.0

In conclusion, the formation energy $\Delta_f U$ can be derived from the formation enthalpy $\Delta_f H$ based on Equation **S8**.

$$\Delta_f U° = \Delta_f H° - \Delta n_g RT \qquad \textbf{(Eq. S8)}$$

The outcomes concerning the enthalpy of formation for nitropyrazoles, determined through bomb calorimeter measurements, are summarized in Table **S12**.

Table S12: Thermodynamic characteristics of DNMNAMP (**4**), including combustion enthalpies, change in gas mole, and formation enthalpy and energy.

Compound	$\Delta_c U°$ [J/g]	$\Delta_c U°$ [cal/g]	$\Delta_c H°$ [kJ/mol]	Δn_g	$\Delta_f H°_{(s)}$ [kJ/mol]	$\Delta_f U°_{(s)}$ [kJ/kg]
**DNMNAMP**	−11927.41	−2850.72	−2931	1.75	106.4	414

S5. NMR spectroscopy

Measured ^{15}N and 2D NMR are presented in the figures S11−S12.

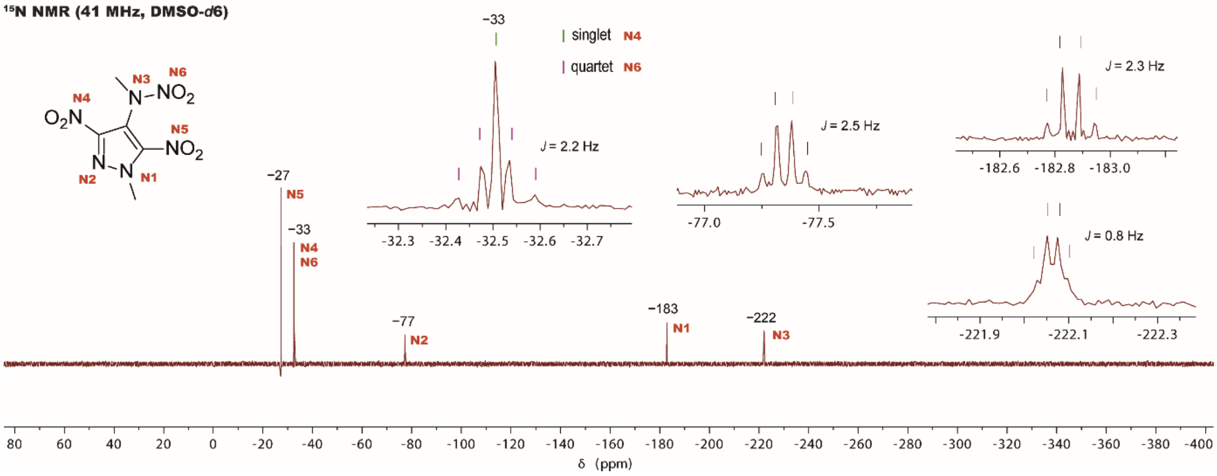

Figure S11: Proton coupled ^{15}N NMR spectrum of compound **4**.

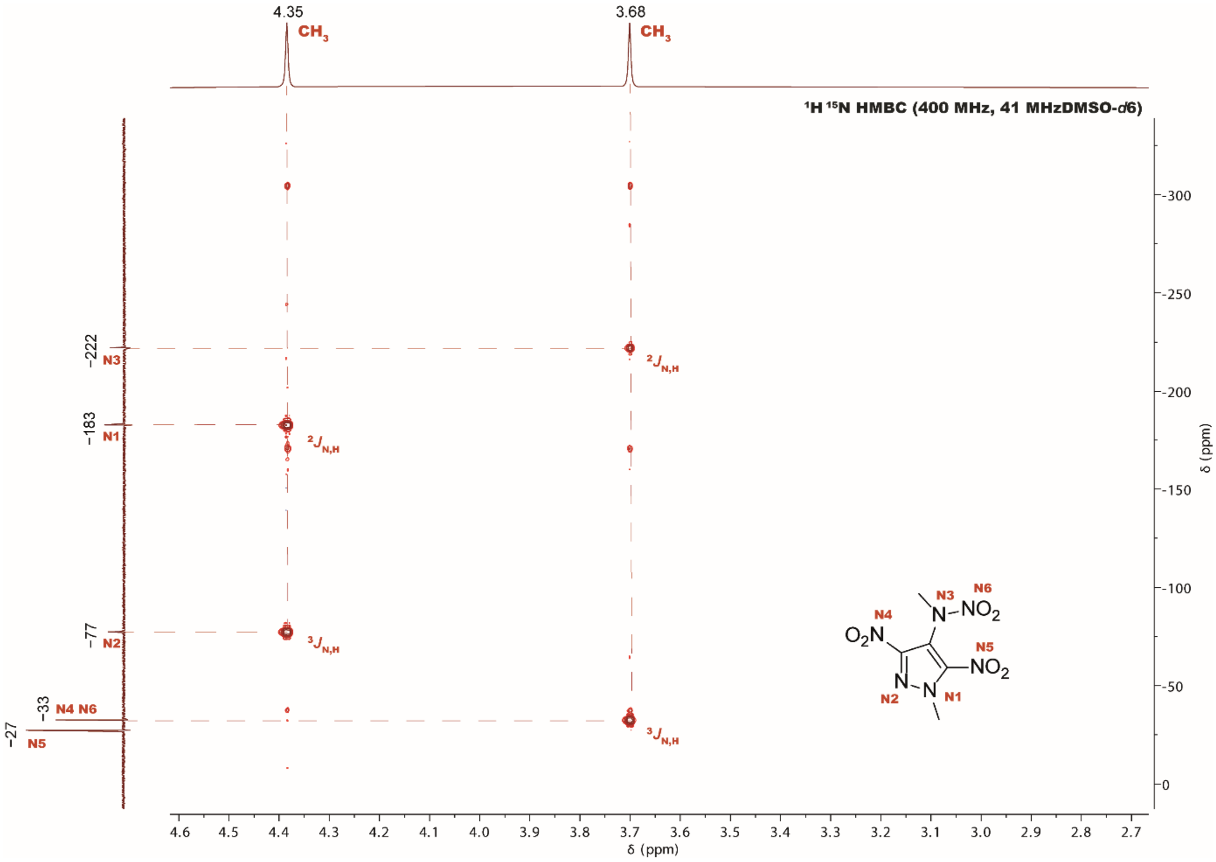

Figure S12: ^{1}H ^{15}N HMBC NMR spectrum of compound **4**.

S6. Thermal Stability

Differential thermal analysis (DTA) was measured on an OZM Research DTA 552-Ex device in a range of 25−400 °C at a heating rate of 5 °C min^{-1}. Thermogravimetric measurements were performed with a Perkin-Elmer TGA 4000 apparatus using a heating rate of 5 °C min^{-1} in a slow stream of nitrogen gas (1mL/min).

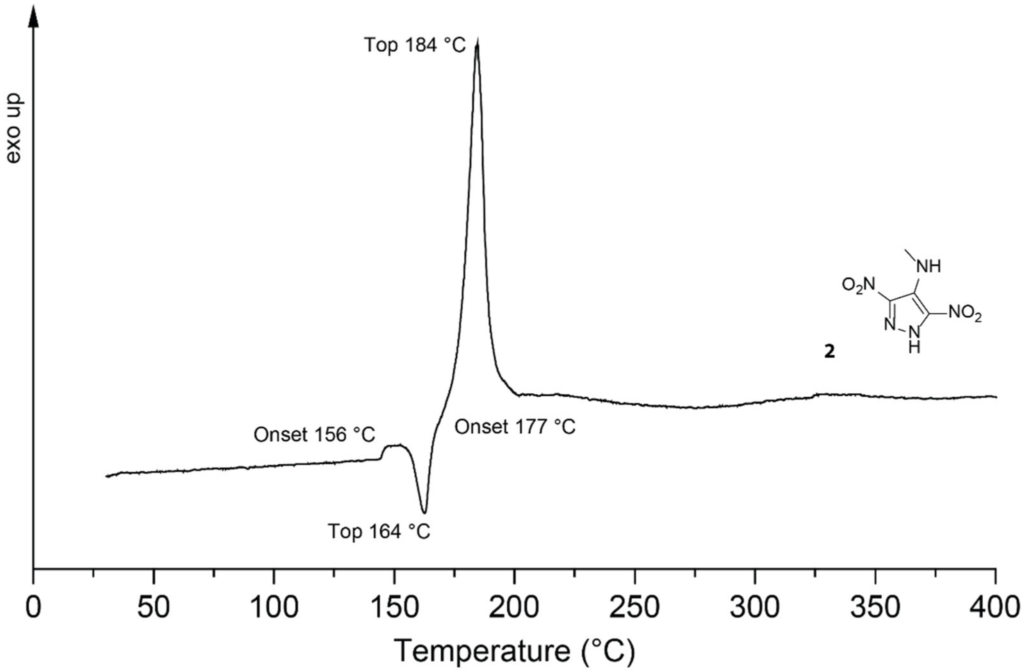

Figure S13: DTA curve of neutral compounds **2** (anhydrous).

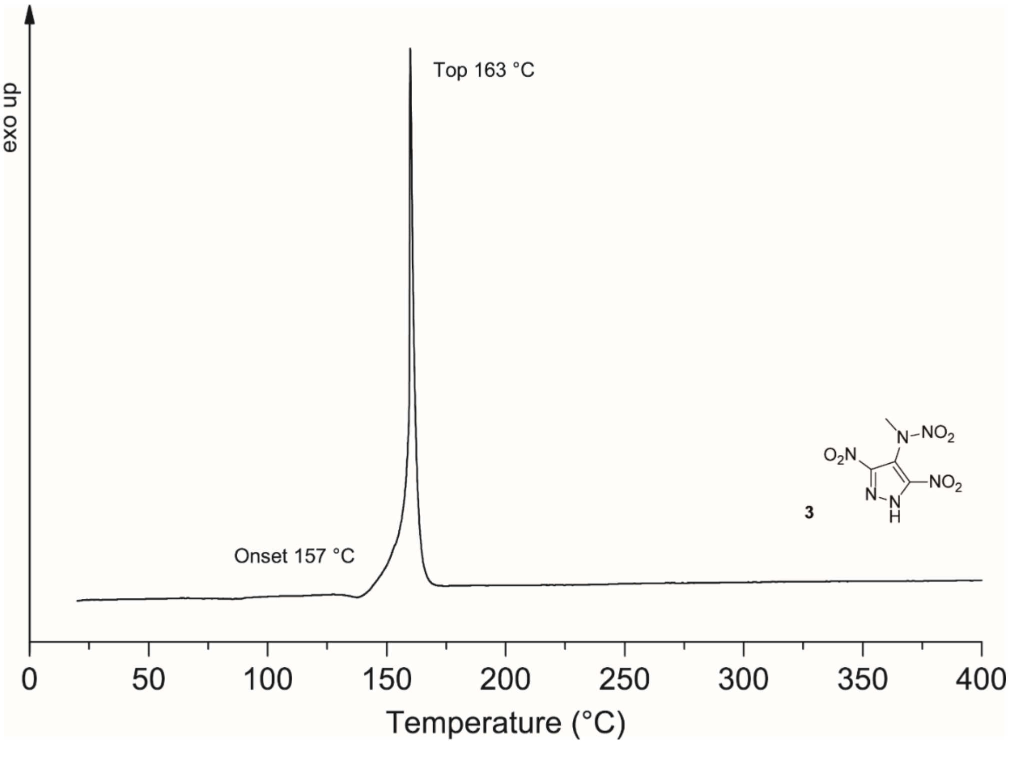

Figure S14: DTA curve of neutral compounds **3**.

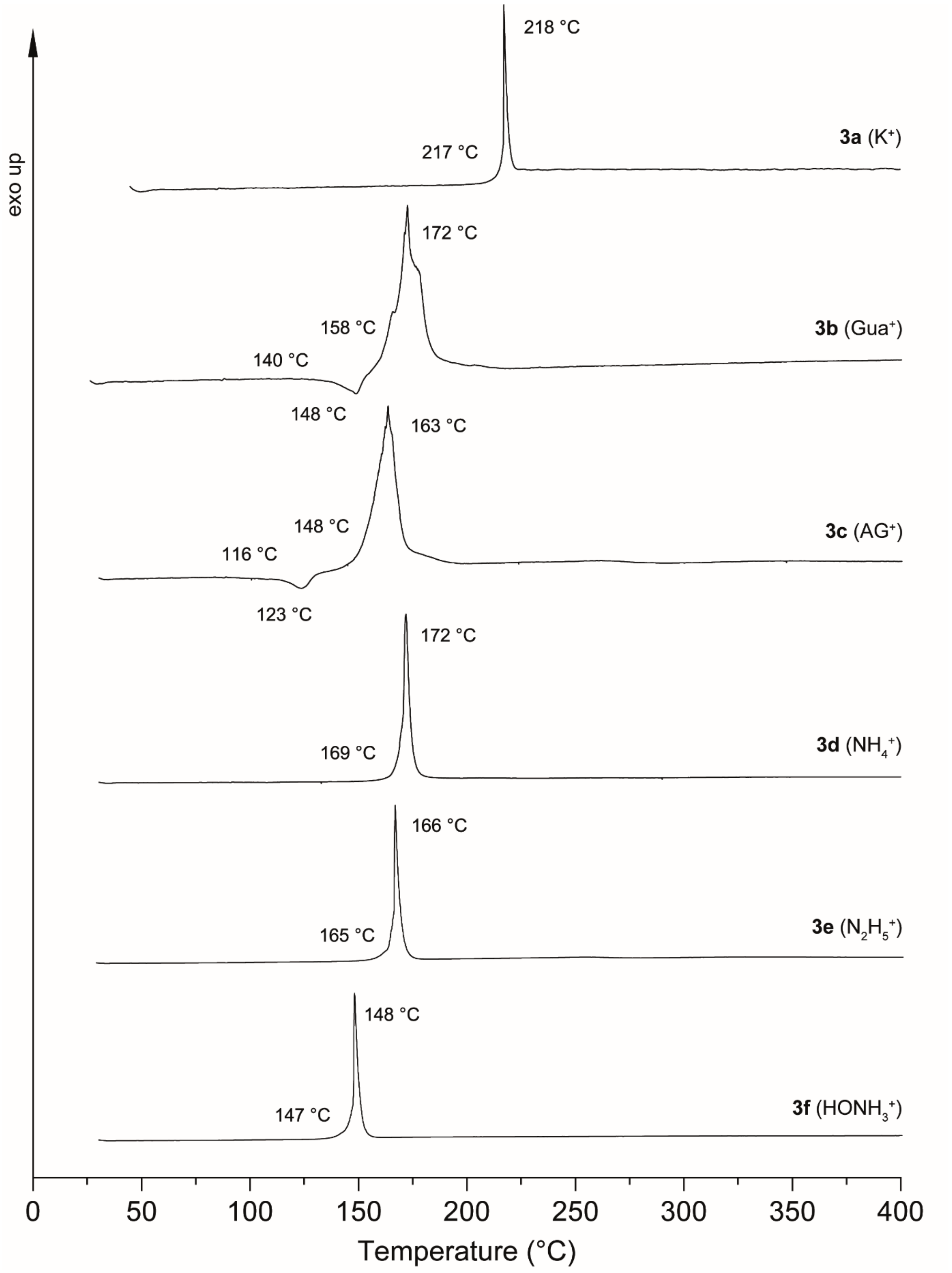

Figure S15: DTA curves of salts of compound **3** (**DNMNAPz**).

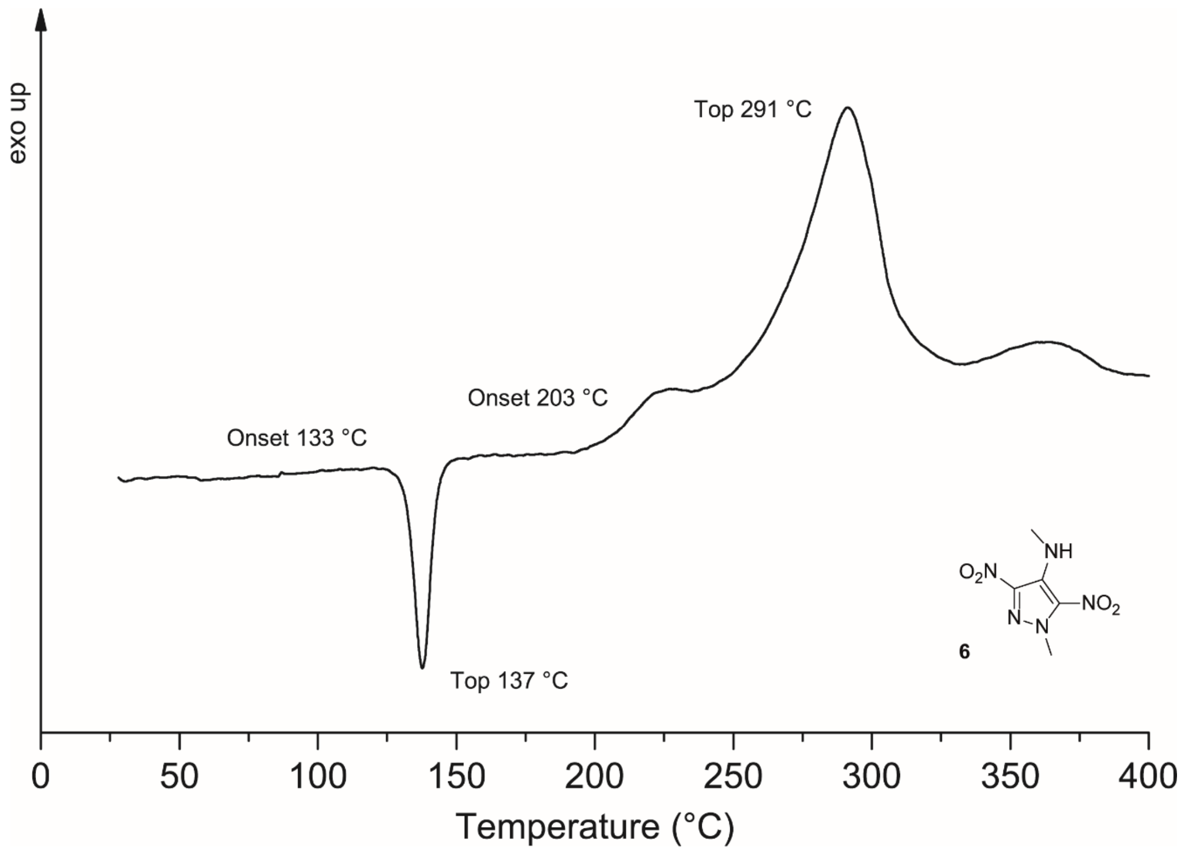

Figure S16: DTA curve of compound **6** (**DNMAMP**).

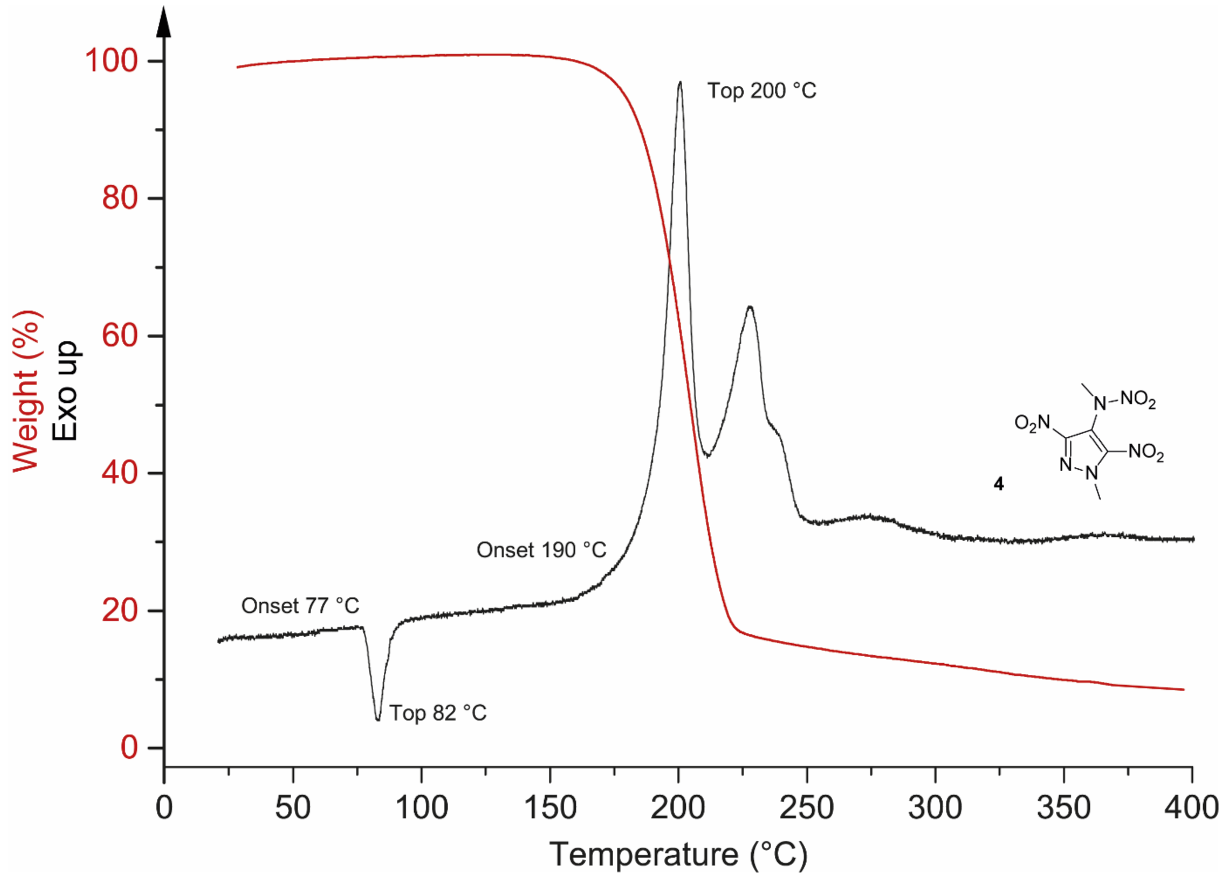

Figure S17: Combined DTA-TGA curve of compound **4** (**DNMNAMPz**).

Compound **4** was subjected to a melting process using a steam bath to evaluate its safe melting characteristics. **Figure S17** illustrates three states of compound **4**: **A** shows the compound in loose powder form before melting, **B** shows compound **4** melted on the steam bath, and **C** shows the solidified compound **4** two days after the melting process. The compound was exposed to the steam bath for a duration of 1 hour. The findings indicate that compound **4** can be safely melted on the steam bath without experiencing decomposition.

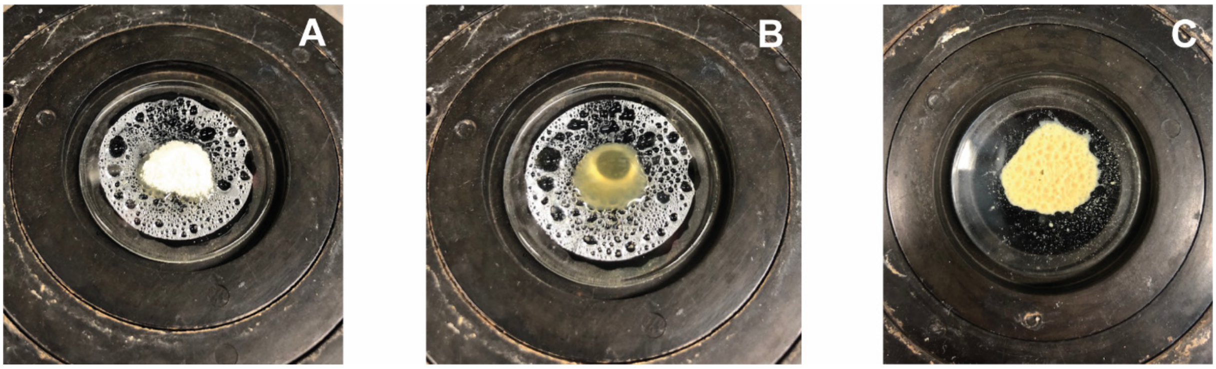

Figure S18: Compound **4** during the melting process: **A** before melting; **B** melting on a steam bath; **C** after 2 days.

S7. SSRT (small-scale shock reactivity test)

To assess the shock reactivity (explosiveness) of the investigated explosives, a small-scale shock reactivity test (SSRT) was conducted. The SSRT is designed to measure the shock reactivity of potentially energetic materials, even below critical diameter, without transitioning to detonation. The test setup combines the advantages of a lead block test[S13] and a gap test.[S14] Each compound was compacted into a perforated steel block. No attenuator (between detonator and sample) or air gap (between sample and aluminum block) was used. Initiation of the tested explosive was achieved using a commercially available detonator (Orica-DYNADET-C2-0ms). Dent sizes resulting from the shock were measured non-contactly using the XYZ-axis motorized 3D profilometer VR-5200, manufactured by Keyence (Osaka, Japan).
More information on the test and its implementation is described by Bauer et al.[S15]

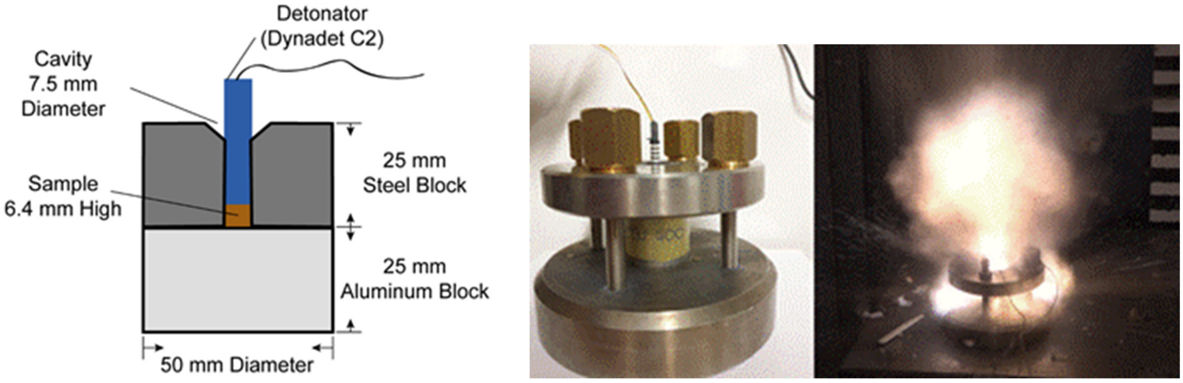

Figure S19: Setup of the SSRT experiment.[S15]

Evaluation of 3,5-dinitro-4-methylnitramino-1-methylpyrazole (4)

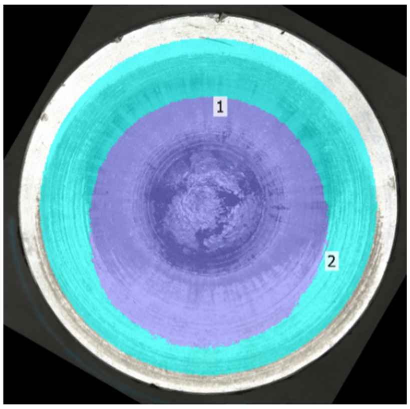

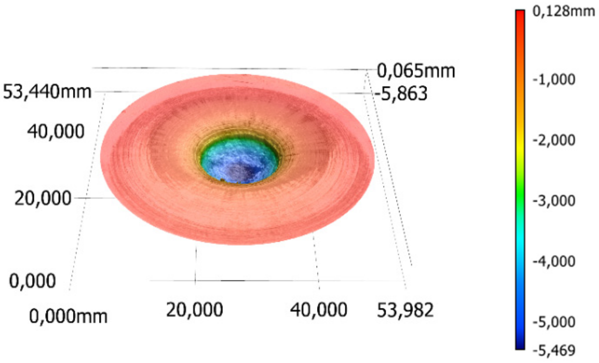

Table S13: SSRT results of **4** (Blast 2).

**3,5-dinitro-4-methylnitramino-1-methylpyrazole<br>Blast 2**	
Measurement 1 [mm³]	1046.82
Measurement 2 [mm³]	1045.40
**Average Volume [mm³]**	**1046.11**

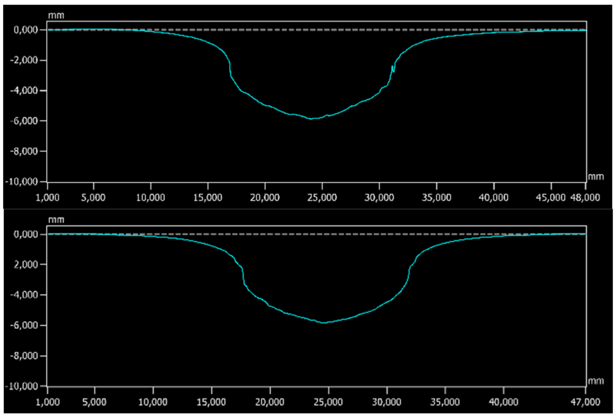

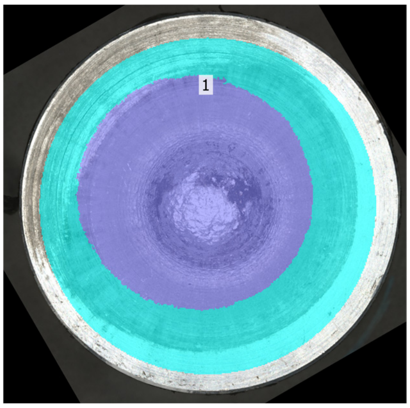

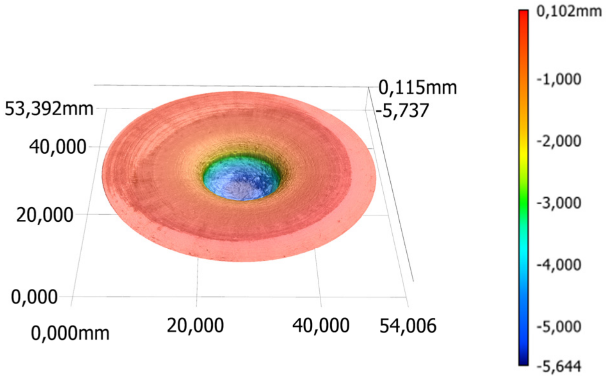

Table S14: SSRT results of **4** (Blast 3).

**3,5-dinitro-4-methylnitramino-1-methylpyrazole<br>Blast 3**	
Measurement 1 [mm³]	1028.65
Measurement 2 [mm³]	1028.48
**Average Volume [mm³]**	**1028.57**

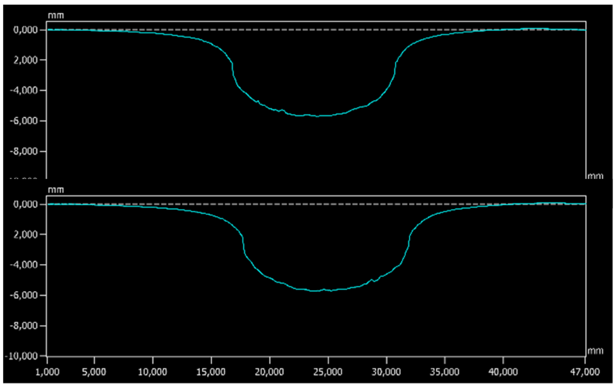

S8. Compatibilities

To ensure the safe handling of energetic substances, it is necessary to consider their thermal stability when they come into contact with other materials. This is particularly important for applications such as explosives and pyrotechnics. In order to assess the compatibility of these substances with various additives, different thermal methods such as differential thermal analysis (DTA), thermogravimetric analysis (TGA), pressure increase in closed systems (PIST), and vacuum stability test (VST) can be utilized. In this study, the DTA method was specifically employed to investigate the compatibility of the energetic substances with different additives (RDX and HMX). Differential thermal analysis (DTA) was measured on an OZM Research DTA 552-Ex device in a range of 25−400 °C at a heating rate of 5 °C min^{-1}. The substances were mixed in the ratio of 1:1.

Compatibilities were evaluated according to the standardized procedure outlined in STANAG 4147.[S16] The analysis involved determining the difference between the endothermic and exothermic maxima of the pure substance and its mixture with the test component. The criteria for determining compatibilities are presented in **Table S15**.

Table S15: Criteria for compatibilities.

temperature difference	description
ΔT ≤ 4 °C	compatible
4 °C < ΔT ≤ 20 °C	moderately
ΔT > 20 °C	incompatible

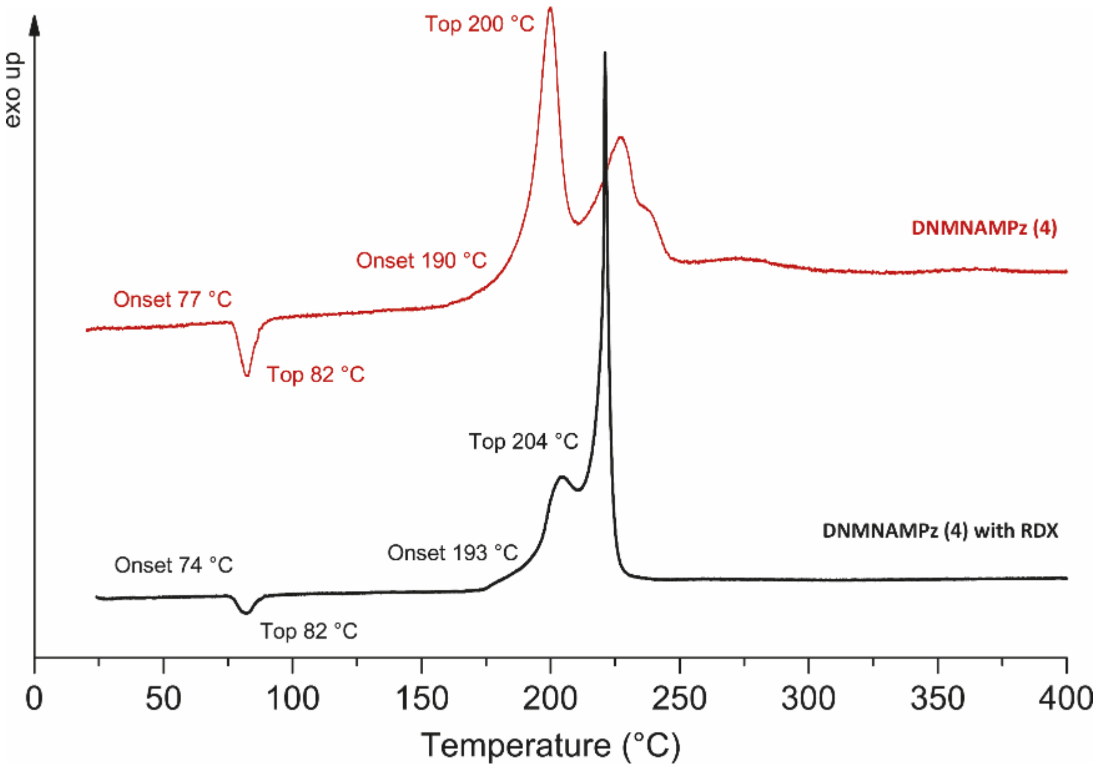

Figure S20: DTA curve of compatibilities measurement of compound **4** with **RDX**.

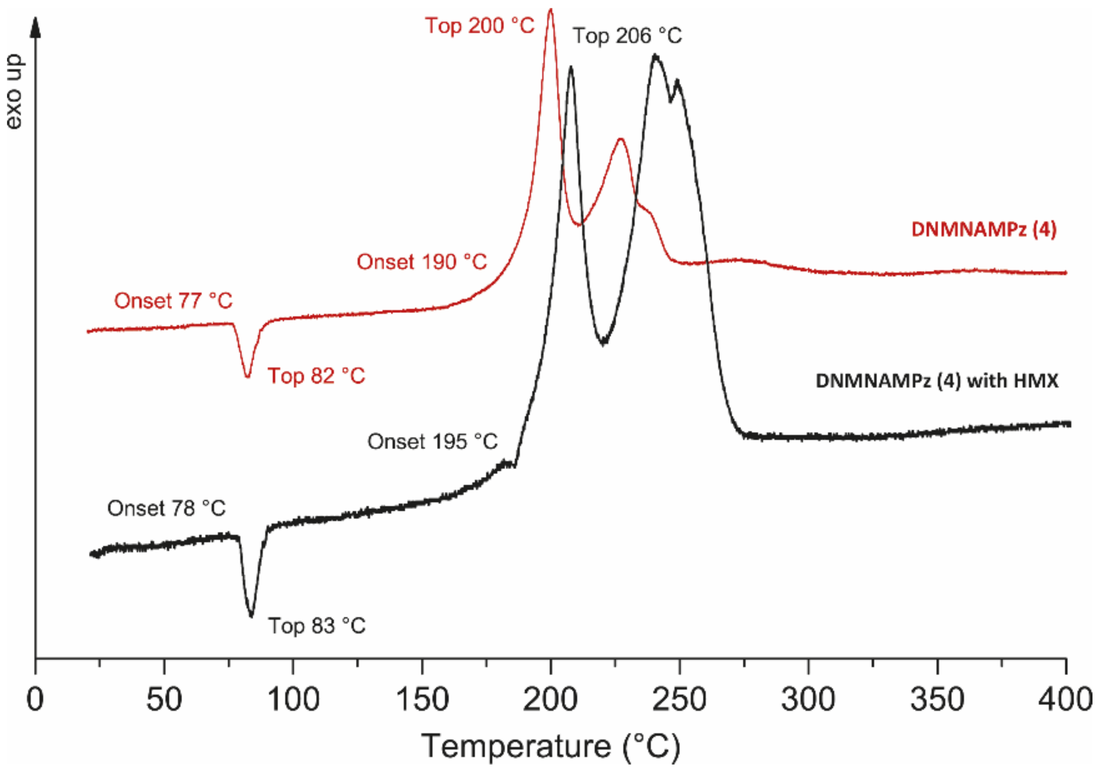

Figure S21: DTA curve of compatibilities measurement of compound **4** with **HMX**.

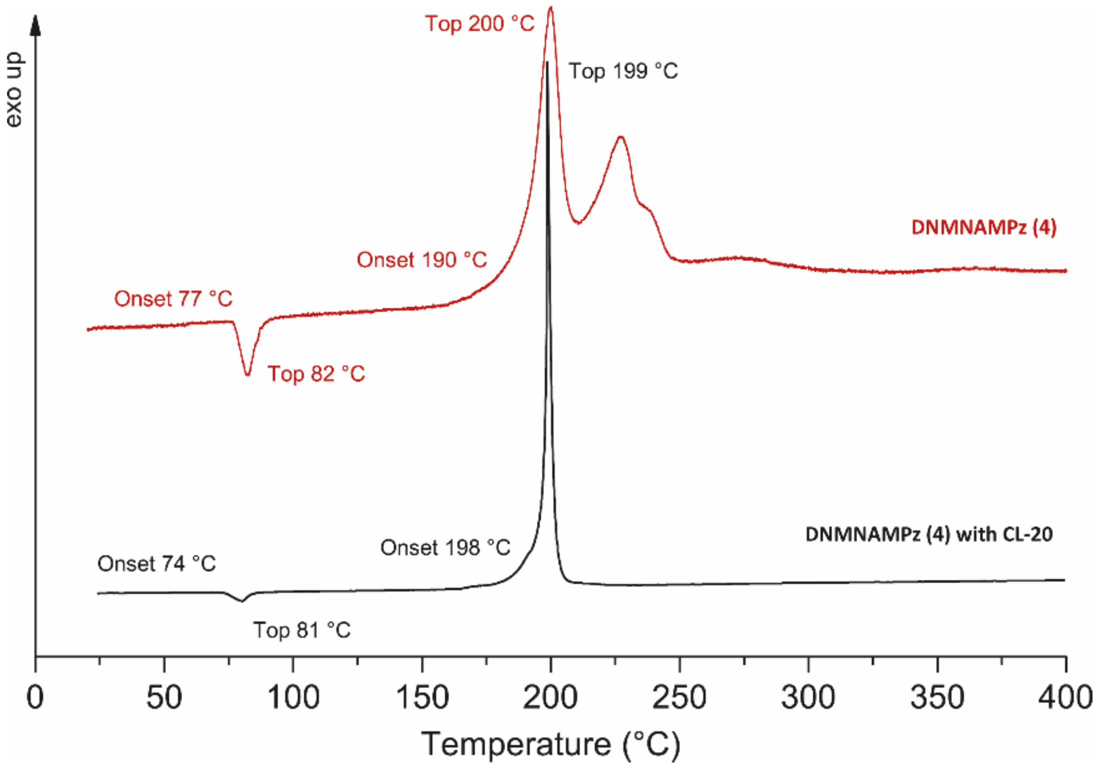

Figure S22: DTA curve of compatibilities measurement of compound **4** with **CL-20**.

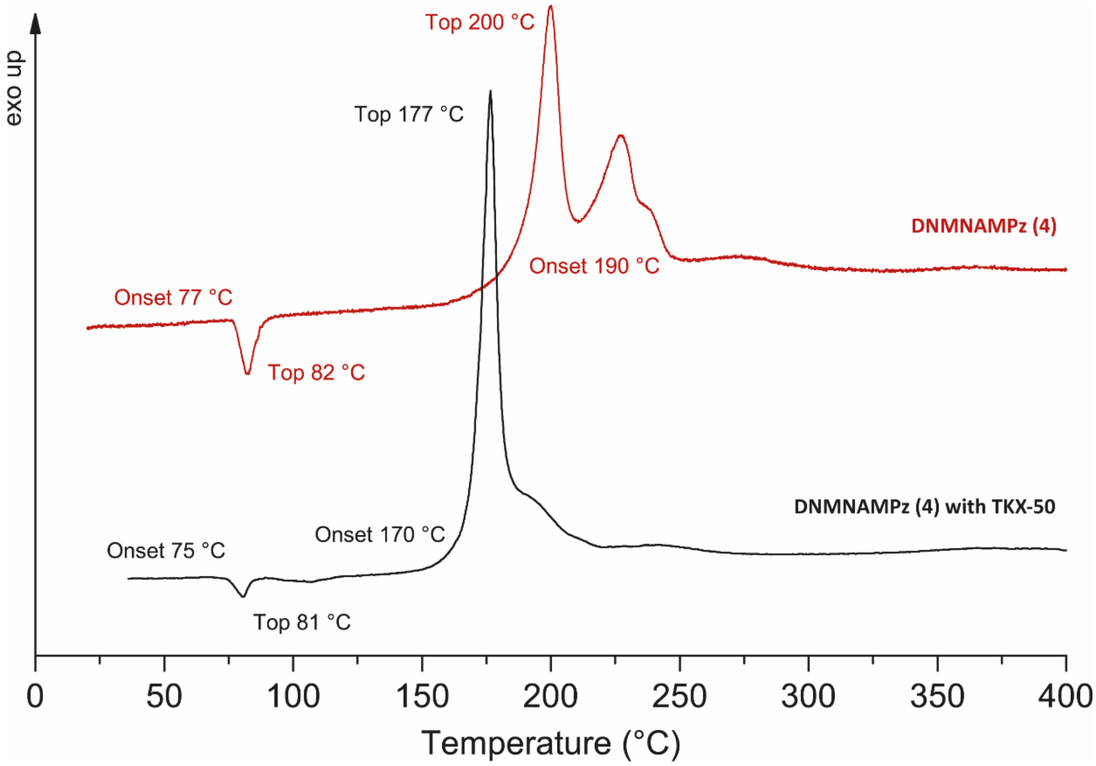

Figure S23: DTA curve of compatibilities measurement of compound **4** with **TKX-20**.

Table S16: Compatibilities of compounds **4** with **RDX**, **HMX** and **CL-20**.

compound	melting [°C]		decompositon [°C]	
	on	top	on	top
**DNMNAMPz (4)**	**77**	**82**	**190**	**200**
DNMNAMPz : RDX (1:1)	74	82	193	204
**difference (RDX)**	**3**	**0**	**3**	**4**
DNMNAMPz : HMX (1:1)	78	83	195	206
**difference (HMX)**	**1**	**1**	**5**	**6**
DNMNAMPz :CL-20 (1:1)	74	81	198	199
**difference (CL-20)**	**3**	**1**	**8**	**1**
DNMNAMPz :TKX-50 (1:1)	75	81	170	177
**difference (TKX-50)**	**2**	**1**	**20**	**23**

S9. AMES Test

The bacterial reverse mutation test, which uses strains of Salmonella typhimurium and Escherichia coli, detects substances that can cause genetic damage and mutations. Point mutations in histidine or tryptophan operons render the test strains incapable of producing these amino acids, making them dependent on external sources. Mutagens reverse these mutations so that the bacteria can grow even in the absence of the required amino acids. The addition of a metabolic system, such as the S9 fraction of rat liver homogenate, allows the identification of mutagens that require metabolic activation to become active. The mutagenic potential of compounds and their metabolites is assessed by exposing bacterial strains to different concentrations of the compounds and evaluating the growth of revertant colonies in the absence of the required amino acids.

The AMES test was carried out by the company Bienta, *Kiev*, *Ukraine*[S17], according the OECD Guidelines for Testing of Chemicals, experiments with the testes agents, positive and negative controls, were conducted in triplicates on six tester strains:

S. typhimurium: TA98, TA100, TA1535, TA1537

E. coli: wp2[pKM101] + wp2 uvrA mixed 1:2

negative control: DMSO

positive control (without metabolic activation): 2-nitrofluorene for TA98 strain, 4-nitroquinoline *N*-oxide for TA100 and E. coli strains, sodium azide for TA1535 strain, 9-aminoacridine for TA1537 strain

positive control (with metabolic activation): 2-aminoantracene

S10. Experimental part and general methods

Caution! Nitropyrazoles are potentially energetic materials with sensitivities towards various stimuli. Therefore, meticulous security precautions (safety glass, face shield, earthed equipment and shoes, Kevlar gloves, and ear plugs) have to be applied while synthesizing and handling the described compounds.

All chemical reagents and solvents were employed as received (Sigma-Aldrich, Acros Organics, ABCR, TCI). NMR spectra were recorded on a 400 MHz instrument (*Bruker AV400* or *Bruker AV400TR*) at room temperature. Chemical shifts (δ) are reported in parts per million (ppm) and refer to tetramethylsilane (^{1}H, ^{13}C) and nitromethane (^{14}N, ^{15}N). All spectra were analyzed with the software MestReNOVA 10.0 from Mestrelab Research, S. L. Infrared (IR) spectra were recorded with a Perkin-Elmer Spektrum One FT-IR spectrometer in a rage of 4000−400 cm^{-1}. Transmittance values are qualitatively described as "very strong" (vs), "strong" (s), "medium" (m), "weak" (w) and "very weak" (vw). Elemental analysis was carried out using an Elementar Vario el or Vario micro by pyrolysis of the sample and subsequent analysis of formed gases (allowed deviation for liquids: +/- 0.5%; for solids: +/- 0.3%). High-resolution mass spectra were recorded with a *Thermo Finnigan LTQ FT-ICR* (ESI) and a *Thermo Finnigan MAT 95* (EI) mass spectrometer. Melting and decomposition points were determined in a rage of 25−400 °C at a heating rate of 5 °C min^{-1} through differential thermal analysis (DTA) with an OZM Research DTA 552-Ex instrument. Thermogravimetric measurements were performed with a Perkin-Elmer TGA 4000 apparatus using a heating rate of 5 °C min^{-1} in a slow stream of nitrogen gas (1mL/min). The sensitivity data were determined using a BAM (Bundesanstalt für Materialforschung) drophammer[S18] according to STANAG 4489[S19] using a modified instruction[S20] and a BAM friction tester[S21] according to STANAG 4487[S22] using a modified instruction. The electrostatic sensitivity test was carried out using the OZM Electric Spark XSpark10 device[S23]. The classification is based on the 'UN Recommendations on the Transport of Dangerous Goods'.[S24]

3,5-Dinitro-4-methylaminopyrazole (2): To a solution of 4-chloro-3,5-dinitropyrazole[S25] (**1**) (3.50 g, 18.2 mmol, 1.0 eq.) in water (20 mL) methylamine (40 wt.% in H_2O, 18 mL, 209 mmol, 11.5 eq.) was added. The mixture was heated at 105 °C overnight. Afterwards the reaction was allowed to cool down to room temperature and was acidified with conc. HCl to p*H* = 1−2. The suspension was filtered, washed with ice-cold H_2O and dried on air to give 3,5-dinitro-4-methylaminopyrazole monohydrate (**2**) (3.36 g, 16.36 mmol) in 90 % yield as a yellow powder.

^{1}H NMR (400 MHz, DMSO-*d*6, 25 °C): δ (ppm) = 7.49 (br, 1H), 3.03 (s, 3H); **^{13}C{^{1}H} NMR** (101 MHz, DMSO-*d*6, 25 °C): δ (ppm) = 138.8, 129.6, 33.4; **^{14}N{^{1}H} NMR** (29 MHz, DMSO-*d*6, 25 °C): δ (ppm) = −24; **EA** ($C_4H_5N_5O_4$, 205.13 g/mol) calc. (found): C 23.42 (24.68), H 3.44 (3.02), N 34.14 (35.96) %; **HRMS** (EI+): *m/z* calculated for $C_4H_5N_5O_4$ [M]: 187.0342, found: 187.0335; **IR** (ATR, rel. int.): $\tilde{\nu}$ (cm^{-1}) = 3395 (s), 3354 (m), 1615 (s), 1558 (m), 1538 (m), 1533 (m), 1498 (s), 1477 (s), 1427 (s), 1380 (s), 1346 (s), 1332 (s), 1299 (vs), 1260 (s), 1166 (m), 1145 (m), 1116 (m), 1063 (w), 1040 (w), 985 (m), 935 (m), 832 (s), 758 (m), 746 (m), 727 (w), 667 (w), 643 (w), 589 (s), 525 (m), 509 (m), 492 (m), 427 (w).

3,5-Dinitro-4-methylnitraminopyrazole (3)[S25]: 3,5-Dinitro-4-methylaminopyrazole monohydrate (**2**) (9.50 g, 46.3 mmol, 1.0 eq.) was added portion wise to TFA (100 mL) at 0 °C. Then fuming HNO_3 (33.0 mL, 79.1 mmol, 1.7 eq.) was added dropwise to the mixture. Afterwards Ac_2O (10.0 mL, 106 mmol, 2.3 eq.) was added slowly and the mixture was stirred at 0 °C for 3 h. The reaction mixture was poured on ice and extracted with EtOAc (3 × 200 mL).

The combined organic extracts were dried over $MgSO_4$, filtered and concentrated under reduced pressure. Crystallization from EtOAc gave 3,5-dinitro-4-methylnitraminopyrazole (**3**) (10.30 g, 44.37 mmol) in 96 % yield in form of yellow crystals.

DTA (5 °C min^{-1}) T_{dec} = 157 °C; **^{1}H NMR** (400 MHz, DMSO-*d*6, 25 °C): δ (ppm) = 9.62 (s, 1H), 3.63 (s, 3H); **^{13}C{^{1}H} NMR** (101 MHz, DMSO-*d*6, 25 °C): δ (ppm) = 147.5, 111.4, 40.5; **^{14}N{^{1}H} NMR** (29 MHz, DMSO-*d*6, 25 °C): δ (ppm) = −24, −31; **EA** ($C_4H_4N_6O_6$, 232.11 g/mol) calc. (found): C 20.70 (20.44), H 1.74 (1.80), N 36.21 (34.96) %. **HRMS** (ESI−): *m/z* calculated for $C_4H_3N_6O_6$ $[M]^-$: 231.0120, found: 231.01204; **IR** (ATR, rel. int.): $\tilde{\nu}$ (cm^{-1}) = 3325 (s), 1739 (w), 1614 (m), 1578 (m), 1539 (s), 1527 (s), 1493 (s), 1472 (m), 1456 (m), 1425 (s), 1389 (m), 1356 (s), 1336 (s), 1320 (s), 1282 (vs), 1192 (m), 1167 (m), 1101 (s), 1005 (m), 944 (m), 840 (vs), 826 (s), 776 (w), 760 (m), 753 (m), 692 (s), 664 (s), 655 (s), 616 (s), 595 (s), 556 (m), 530 (w), 510 (m), 425 (vw), 405 (vw).

General synthesis of 3,5-dinitro-4-methylnitraminopyrazolate salts (3a–f)

To a solution of 3,5-dinitro-4-methylnitraminopyrazole **(3)** (0.30 g, 1.29 mmol, 1.0 eq.) in ethanol (20 ml) the corresponding base (**K^+:** KOH 72.6 mg, 1.29 mmol; **Gua^+:** guanidinium carbonate 233 mg, 1.29 mmol; **AG^+:** aminoguanidinium bicarbonate 176 mg, 1.29 mmol; **NH_4^+:** $(NH_4)_2CO_3$., 147 mmol, 1.29 mmol; **$N_2H_5^+$:** N_2H_4•H_2O 0.13 mL, 2.58 mmol; **NH_3OH^+:** NH_2OH 50% aq., 0.16 mL, 2.58 mmol) was added. The reaction mixtures were stirred for 10 min at 80 °C and left for crystallization.

Potassium 3,5-dinitro-4-methylnitraminopyrazolate (3a)

Yield: (0.29 g, 1.08 mmol, 84%) as yellow crystals.

DTA (5 °C min^{-1}) T_{dec} = 217 °C; **^{1}H NMR** (400 MHz, DMSO-*d*6, 25 °C): δ (ppm) = 3.62 (s, 3H); **^{13}C{^{1}H} NMR** (101 MHz, DMSO-*d*6, 25 °C): δ (ppm) = 148.9, 111.2, 40.6; **^{14}N{^{1}H} NMR** (29 MHz, DMSO-*d*6, 25 °C): δ (ppm) = −21, −31; **EA** ($C_4H_3KN_6O_6$, 270.20 g/mol) calc. (found): C 17.78 (17.69), H 1.12 (1.25), N 31.10 (30.48) %; **HRMS** (EI+): *m/z* calculated for $C_4H_3N_6O_6$ $[M]^-$: 231.1045, found: 231.01195; **IR** (ATR, rel. int.): $\tilde{\nu}$ (cm^{-1}) = 1738 (w), 1652 (w), 1633 (w), 1594 (m), 1529 (m), 1495 (vs), 1456 (m), 1434 (s), 1418 (s), 1396 (m), 1362 (s), 1344 (s), 1300 (vs), 1263 (s), 1218 (m), 1180 (m), 1148 (m), 1113 (m), 1005 (m), 968 (m), 869 (w), 842 (vs), 831 (s), 764 (m), 753 (m), 689 (s), 678 (m), 660 (m), 602 (m), 412 (m).

Guanidinium 3,5-dinitro-4-methylnitraminopyrazolate (3b)

Yield: (0.28 g, 0.97 mmol, 75%) as yellow crystals.

DTA (5 °C min^{-1}) T_{melt} = 140 °C, T_{dec} = 158 °C; **^{1}H NMR** (400 MHz, DMSO-*d*6, 25 °C): δ (ppm) = 6.54 (br, 6H), 3.60 (s, 3H); **^{13}C{^{1}H} NMR** (101 MHz, DMSO-*d*6, 25 °C): δ (ppm) = 157.9, 149.3, 111.1, 40.5; **^{14}N{^{1}H} NMR** (29 MHz, DMSO-*d*6, 25 °C): δ (ppm) = −18, −30; **EA** ($C_5H_9N_9O_6$, 291.18 g/mol) calc. (found): C 20.29 (20.74), H 3.12 (2.82), N 43.29 (43.24) %; **HRMS** (EI+): *m/z* calculated for $C_4H_3N_6O_6$ $[M]^-$: 231.1045, found: 231.01197; **IR** (ATR, rel. int.): $\tilde{\nu}$ (cm^{-1}) = 3454 (w), 3420 (w), 3390 (m), 2971 (m), 1739 (vs), 1643 (m), 1591 (m), 1495 (s), 1455 (m), 1440 (m), 1420 (s), 1365 (vs), 1350 (vs), 1319 (s), 1291 (s), 1262 (m), 1229 (s), 1217 (s), 1207 (s), 1157 (m), 1105 (m), 1006 (m), 954 (w), 845 (s), 831 (m), 763 (m), 693 (m), 665 (w), 529 (m), 514 (m), 492 (m), 423 (w).

Aminoguanidinium 3,5-dinitro-4-methylaminopyrazolate (3c)

Yield: (0.28 g, 0.93 mmol, 72%) as orange crystals.

DTA (5 °C min^{-1}) T_{melt} = 116 °C, T_{dec} = 148 °C; **^{1}H NMR** (400 MHz, DMSO-*d*6, 25 °C): δ (ppm) = 8.54 (br, 1H), 7.12 (br, 4H), 4.67 (s, 2H), 3.61 (s, 3H); **^{13}C{^{1}H} NMR** (101 MHz, DMSO-*d*6, 25 °C): δ (ppm) = 158.9, 149.4, 111.2, 40.5; **^{14}N{^{1}H} NMR** (29 MHz, DMSO-*d*6, 25 °C): δ (ppm) = −19, −30; **EA** ($C_5H_{10}N_{10}O_6$, 306.20 g/mol) calc. (found): C 19.61 (19.03), H 3.29 (3.28), N 45.74 (44.76) %; **HRMS** (EI+): *m/z* calculated for $C_4H_3N_6O_6$ [M]$^-$: 231.1045, found: 231.01196; **IR** (ATR, rel. int.): $\tilde{\nu}$ (cm^{-1}) = 3465 (m), 3427 (m), 3368 (m), 3200 (m), 3016 (w), 2860 (w), 1739 (m), 1729 (w), 1657 (s), 1590 (m), 1511 (m), 1490 (s), 1451 (m), 1421 (s), 1361 (s), 1304 (vs), 1265 (s), 1217 (m), 1200 (m), 1156 (m), 1102 (m), 1007 (m), 962 (m), 948 (m), 867 (w), 841 (s), 764 (m), 753 (m), 716 (m), 693 (s), 661 (m), 616 (w), 562 (w), 496 (m), 408 (m).

Ammonium 3,5-dinitro-4-methylaminopyrazolate (3d)

Yield: (0.23 g, 0.93 mmol, 80%) as yellow crystals.

DTA (5 °C min^{-1}) T_{dec} = 169 °C; **^{1}H NMR** (400 MHz, DMSO-*d*6, 25 °C): δ (ppm) = 7.12 (s, 4H), 3.61 (s, 3H); **^{13}C{^{1}H} NMR** (101 MHz, DMSO-*d*6, 25 °C): δ (ppm) = 149.4, 111.2, 40.6; **^{14}N{^{1}H} NMR** (29 MHz, DMSO-*d*6, 25 °C): δ (ppm) = −18, −30, −359; **EA** ($C_4H_7N_7O_6$, 249.14 g/mol) calc. (found): C 19.28 (19.19), H 2.83 (2.58), N 39.35 (39.08) %; **HRMS** (EI+): *m/z* calculated for $C_4H_3N_6O_6$ [M]$^-$: 231.1045, found: 231.01198; **IR** (ATR, rel. int.): $\tilde{\nu}$ (cm^{-1}) = 3228 (m), 3016 (m), 2798 (m), 1672 (w), 1591 (m), 1498 (s), 1456 (m), 1417 (s), 1408 (s), 1358 (s), 1344 (vs), 1318 (vs), 1289 (vs), 1260 (vs), 1167 (m), 1144 (m), 1123 (m), 1109 (s), 1073 (m), 1062 (m), 1009 (m), 981 (w), 846 (s), 828 (m), 762 (m), 750 (s), 694 (m), 659 (s), 607 (m), 561 (w), 406 (w).

Hydrazinium 3,5-dinitro-4-methylaminopyrazolate (3e)

Yield: (0.22 g, 0.83 mmol, 64%) as yellow crystals.

DTA (5 °C min^{-1}) T_{dec} = 165 °C; **^{1}H NMR** (400 MHz, DMSO-*d*6, 25 °C): δ (ppm) = 6.23 (br, 6H), 3.60 (s, 3H); **^{13}C{^{1}H} NMR** (101 MHz, DMSO-*d*6, 25 °C): δ (ppm) = 149.4, 111.3, 40.6; **^{14}N{^{1}H} NMR** (29 MHz, DMSO-*d*6, 25 °C): δ (ppm) = −19, −30; **EA** ($C_4H_8N_8O_6$, 264.16 g/mol) calc. (found): C 18.19 (18.34), H 3.05 (2.96), N 42.42 (42.25) %; **HRMS** (EI+): *m/z* calculated for $C_4H_3N_6O_6$ [M]$^-$: 231.1045, found: 231.01195; **IR** (ATR, rel. int.): $\tilde{\nu}$ (cm^{-1}) = 3346 (w), 3227 (w), 3184 (w), 2591 (m), 1636 (w), 1591 (m), 1532 (m), 1495 (s), 1449 (m), 1418 (s), 1352 (s), 1317 (vs), 1281 (vs), 1264 (s), 1188 (m), 1167 (m), 1099 (s), 1015 (m), 946 (m), 934 (m), 846 (vs), 830 (m), 762 (m), 752 (m), 694 (m), 673 (m), 653 (m), 600 (w), 570 (w), 447 (w), 407 (w).

Hydroxylammonium 3,5-dinitro-4-methylaminopyrazolate (3f)

Yield: (0.24 g, 0.89 mmol, 69%) as orange crystals.
DTA (5 °C min^{-1}) T_{dec} = 147 °C; **^{1}H NMR** (400 MHz, DMSO-*d*6, 25 °C): δ (ppm) = 9.99 (br, 3H), 3.62 (s, 3H); **^{13}C{^{1}H} NMR** (101 MHz, DMSO-*d*6, 25 °C): δ (ppm) = 149.4, 111.2, 40.6; **^{14}N{^{1}H} NMR** (29 MHz, DMSO-*d*6, 25 °C): δ (ppm) = −18, −30; **EA** ($C_4H_7N_7O_7$, 265.14 g/mol) calc. (found): C 18.12 (18.05), H 2.66 (2.46), N 36.98 (36.64) %; **HRMS** (EI+): *m/z* calculated for

$C_4H_3N_6O_6$ [M]⁻: 231.1045, found: 231.01196; **IR** (ATR, rel. int.): $\tilde{\nu}$ (cm^{-1}) = 3266 (w), 3187 (m), 2825 (m), 2601 (m), 1595 (m), 1532 (m), 1497 (s), 1450 (m), 1421 (vs), 1357 (vs), 1321 (vs), 1282 (vs), 1262 (s), 1210 (s), 1173 (m), 1107 (s), 1062 (m), 1022 (m), 1006 (m), 997 (m), 952 (w), 879 (vw), 849 (vs), 830 (m), 764 (m), 751 (m), 704 (m), 693 (m), 675 (m), 666 (m), 650 (m), 601 (w), 571 (m), 527 (vw), 408 (m).

3,5-Dinitro-4-methylnitramino-1-methylpyrazole (4): 3,5-Dinitro-4-methylnitraminopyrazole (**2**) (1.50 g, 6.46 mmol, 1.00 eq.) was added to a solution of sodium bicarbonate (1.00 g, 11.9 mmol, 1.84 eq.) in water (20 ml). After 10 min dimethyl sulfate (0.68 mL, 7.17 mmol, 1.10 eq.) was added. The reaction mixture was stirred at room temperature for 1.5 h. Formed precipitation was filtered off and dried on air to give 3,5-dinitro-4-methylnitramino-1-methylpyrazole (**4**) (1.10 g, 4.20 mmol) in 69 % yield as an off-white powder.

DTA (5 °C min^{-1}) T_{melt} = 77 °C, T_{dec} = 190 °C; **^{1}H NMR** (400 MHz, DMSO-*d*6, 25 °C): δ (ppm) = 4.35 (s, 3H), 3.68 (s, 3H); **^{13}C{^{1}H} NMR** (101 MHz, DMSO-*d*6, 25 °C): δ (ppm) = 145.2, 140.32, 113.0, 43.2, 40.2; **^{14}N{^{1}H} NMR** (29 MHz, DMSO-*d*6, 25 °C): δ (ppm) = −32; **^{15}N NMR** (41 MHz, DMSO-*d*6, 25 °C): δ (ppm) = −27.2, −32.5, 32.5 (q, $^3J_{N,H}$ = 2.2 Hz), −77.3 (q, $^3J_{N,H}$ = 2.5 Hz), −182.9 (q, $^2J_{N,H}$ = 2.3 Hz), −222.1 (q, $^2J_{N,H}$ = 0.8 Hz); **EA** ($C_5H_6N_6O_6$, 246.14 g/mol) calc. (found): C 24.40 (24.18), H 2.46 (2.65), N 34.14 (34.04) %; **HRMS** (EI+): *m/z* calculated for $C_5H_6N_5O_4^+$ [M+]: 200.0414, found: 200.0417; **IR** (ATR, rel. int.): $\tilde{\nu}$ (cm^{-1}) = 1739 (w), 1600 (s), 1563 (m), 1525 (vs), 1462 (m), 1443 (s), 1424 (s), 1382 (m), 1367 (m), 1336 (vs), 1312 (m), 1286 (vs), 1258 (s), 1218 (m), 1170 (w), 1145 (m), 1123 (w), 1094 (m), 1056 (m), 954 (m), 884 (s), 830 (m), 784 (s), 754 (m), 729 (m), 691 (m), 666 (s), 628 (w), 598 (w), 486 (w), 431 (w).

4-Chloro-3,5-dinitro-1-methylpyrazole (5)[S25]: 4-Chlor-3,5-dinitropyrazole (**1**) (0.58 g, 3.00 mmol, 1.0 eq.) was added to a solution of sodium bicarbonate (0.51 g, 6.00 mmol, 2.0 eq.) in water (10 ml). After 10 min dimethyl sulfate (0.34 mL, 3.60 mmol, 1.2 eq.) was added. The reaction mixture was stirred at room temperature for 4 h. Formed precipitation was filtered off and dried on air to give 4-chloro-3,5-dinitro-1-methyl-pyrazole (**5**) (0.42 g, 2.03 mmol) in 67 % yield as an colourless powder.

DTA (5 °C min^{-1}) T_{endo} = 81 °C; **^{1}H NMR** (400 MHz, DMSO-*d*6, 25 °C): δ (ppm) = 4.28 (s, 3H); **^{13}C{^{1}H} NMR** (101 MHz, DMSO-*d*6, 25 °C): δ (ppm) = 147.7, 142.8, 105.6, 43.5; **^{14}N{^{1}H} NMR** (29 MHz, DMSO-*d*6, 25 °C): δ (ppm) = −28, −33; **EA** ($C_4H_3ClN_4O_4$, 205:54 g/mol) calc. (found): C 23.26 (23.28), H 1.46 (1.66), N 27.13 (26.97) %; **HRMS** (EI+): *m/z* calculated for $C_4H_3ClN_4O_4$ [M]: 205.9843, found: 205.9834; **IR** (ATR, rel. int.): $\tilde{\nu}$ (cm^{-1}) = 1557 (vs), 1501 (vs), 1434 (vs), 1398 (m), 1375 (m), 1329 (vs), 1303 (vs), 1153 (m), 1124 (w), 1089 (m), 1045 (s), 1018 (w), 878 (s), 836 (m), 823 (m), 760 (s), 750 (s), 675 (m), 622 (m), 546 (w), 481 (m).

3,5-Dinitro-4-methylamino-1-methylpyrazole (6):
Variant A[S26]: 3,5-Dinitro-4-methylaminopyrazole monohydrate (**2**) (0.62 g, 3.00 mmol, 1.0 eq.) was added to a solution of sodium bicarbonate (0.51 g, 6.00 mmol, 2.0 eq.) in water (10 ml). After 10 min dimethyl sulfate (0.34 mL, 3.60 mmol, 1.2 eq.) was added. The reaction mixture was stirred at room temperature for 4 h. Formed precipitation was filtered off and dried on air to give 3,5-dinitro-4-methylamino-1-methylpyrazole (**6**) (0.48 g, 2.40 mmol) in 80 % yield as a yellow powder.
Variant B: To a solution of 4-chloro-3,5-dinitro-1-methylpyrazole (**5**) (0.62 g, 3.00 mmol, 1.0 eq) in DMSO (3 mL) CH_3NH_2 (40 wt.% in H_2O, 0.7 mL, 9.00 mmol, 3.0 eq.) was added. The

mixture was heated at 110 °C for 2 h. Afterwards the reaction was allowed to cool down to room temperature and poured in ice-water. The precipitation was filtered, washed with ice-cold H_2O and dried on air to give 3,5-dinitro-4-methylamino-1-methylpyrazole (**6**) (0.45 g, 2.25 mmol) in 75 % yield as a yellowish powder.

DTA (5 °C min^{-1}) T_{melt} = 133 °C, T_{dec} = 203 °C; **^{1}H NMR** (400 MHz, DMSO-*d*6, 25 °C): δ (ppm) = 7.26 (q, $^3J_{H,H}$ = 5.3 Hz, 1H), 4.16 (s, 3H), 2.94 (d, $^3J_{H,H}$ = 5.4 Hz, 3H); **^{13}C{^{1}H} NMR** (101 MHz, DMSO-*d*6, 25 °C): δ (ppm) = 140.7, 133.2, 130.8, 43.0, 33.1; **^{14}N{^{1}H} NMR** (29 MHz, DMSO-*d*6, 25 °C): δ (ppm) = −24, −28; **EA** ($C_5H_7N_5O_4$, 201.14 g/mol) calc. (found): C 29.86 (29.80), H 3.51 (3.24), N 34.82 (35.04) %; **HRMS** (EI+): *m/z* calculated for $C_5H_7N_5O_4$ [M]: 201.0498, found: 201.0495; **IR** (ATR, rel. int.): $\tilde{\nu}$ (cm^{-1}) = 3364 (w), 3323 (w), 1737 (vw), 1610 (s), 1516 (m), 1472 (m), 1459 (m), 1435 (s), 1400 (m), 1362 (m), 1330 (s), 1291 (vs), 1217 (s), 1162 (m), 1136 (m), 1069 (m), 1029 (s), 890 (s), 826 (m), 770 (s), 759 (m), 746 (s), 664 (w), 639 (m), 616 (m), 599 (m), 484 (w).

S11. References

[S1] CrysAlisPro, Oxford Diffraction Ltd. version 171.33.41, **2009**.

[S2] G. M. Sheldrick, *Acta Cryst.*, **2015**, A71, 3–8.

[S3] Dolomanov, O. V., Bourhis, L. J., Gildea, R. J., Howard, J. A. K. & Puschmann, H. J., *Appl. Cryst.*, **2009**, 42, 339–341.

[S4] SCALE3 ABSPACK – An Oxford Diffraction program (1.0.4, gui: 1.0.3), Oxford Diffraction Ltd., **2005**.

[S5] APEX3. Bruker AXS Inc., Madison, Wisconsin, USA.

[S6] M. J. Frisch, G. W. Trucks, H. B. Schlegel, G. E. Scuseria, M. A. Robb, J. R. Cheeseman, G. Scalmani, V. Barone, G. A. Petersson, H. Nakatsuji, X. Li, M. Caricato, A. V. Marenich, J. Bloino, B. G. Janesko, R. Gomperts, B. Mennucci, H. P. Hratchian, J. V. Ortiz, A. F. Izmaylov, J. L. Sonnenberg, D. Williams-Young, F. Ding, F. Lipparini, F. Egidi, J. Goings, B. Peng, A. Petrone, T. Henderson, D. Ranasinghe, V. G. Zakrzewski, J. Gao, N. Rega, G. Zheng, W. Liang, M. Hada, M. Ehara, K. Toyota, R. Fukuda, J. Hasegawa, M. Ishida, T. Nakajima, Y. Honda, O. Kitao, H. Nakai, T. Vreven, K. Throssell, J. A. Montgomery, Jr., J. E. Peralta, F. Ogliaro, M. J. Bearpark, J. J. Heyd, E. N. Brothers, K. N. Kudin, V. N. Staroverov, T. A. Keith, R. Kobayashi, J. Normand, K. Raghavachari, A. P. Rendell, J. C. Burant, S. S. Iyengar, J. Tomasi, M. Cossi, J. M. Millam, M. Klene, C. Adamo, R. Cammi, J. W. Ochterski, R. L. Martin, K. Morokuma, O. Farkas, J. B. Foresman, and D. J. Fox, Gaussian16 ,Gaussian, Inc., Wallingford, CT, USA, **2016**.

[S7] a) J. W. Ochterski, G. A. Petersson, J. A. Montgomery, *J. Chem. Phys*. **1996**, *104*, 2598–2619; b) J. A. Montgomery, M. J. Frisch, J. W. Ochterski, G. A. Petersson, *J. Chem. Phys*. **2000**, *12*, 6532–6542.

[S8] a) L. A. Curtiss, K. Raghavachari, P. C. Redfern, J. A. Pople, *J. Chem. Phys*. **1997**, *106*, 1063–1079; b) B. M. Rice, S. V. Pai, J. Hare, *Combust. Flame* **1999**, *118*, 445–458; c) E. F. C. Byrd, B. M. Rice, *J. Phys. Chem. A* **2006**, *110*, 1005–1013.

[S9] P. J. Linstrom, W. G. Mallard (Editors), *National Institute of Standards and Technology Standard Reference Database Number 69*, Gaithersburg MD, 20899, http://webbook.nist.gov/chemistry/ (June **2025**).

[S10] a) F. Trouton, Philos Mag (*1876-1900*) **1884**, *18*, 54–57; b) M. S. Westwell, M. S. Searle, D. J. Wales, D. H. Williams, *J. Am. Chem. Soc*. **1995**, *117*, 5013–5015.

[S11] F. D. Rossini, *Experimental Thermochemistry. Measurement of Heats of Reaction*; Interscience Publishers Inc., **1956**.

[S12] E. Paulechka, D. Riccardi, *Combustion Calorimetry Tool – NIST Standard Reference Database 206* National Institute of Standard and Technology, **2019**.

[S13] R. Meyer, J. Köhler, A. Homburg, *Explosives*, 6th edn., Wiley, Weinheim, **2007**, p. 196–198.

[S14] R. Meyer, J. Köhler, A. Homburg, *Explosive*s, 6th edn., Wiley, Weinheim, **2007**, p. 145.

[S15] L. Bauer, M. Benz, T. M. Klapoetke, *Propellants Explos. Pyrotech*. **2022**, *47*, e202100332.

[S16] NATO standardization agreement (STANGAG), *Chemical compatibility of ammunition components with explosives (non-nuclear application)*, No. 4147 2. ed., **2001**.

[S17] Bienta, https://bienta.net (June **2025**).

[S18] http://www.bam.de (June **2025**).

[S19] NATO Standardization Agreement (STANAG) on Explosives, *Impact Sensitivity Tests*, no. 4489, 1st ed., September 17, **1999**.

[S20] WIWEB-Standardarbeitsanweisung 4-5.1.02, Ermittlung der Explosionsgefährlichkeit, hier der Schlagempfindlichkeit mit dem Fallhammer, November 8, **2002**.

[S21] NATO Standardization Agreement (STANAG) on Explosives, *Friction Sensitivity Tests*, no. 4487, 1st ed., August 22, **2002**.

[S22] WIWEB-Standardarbeitsanweisung 4-5.1.03, Ermittlung der Explosionsgefährlichkeit oder der Reibeempfindlichkeit mit dem Reibeapparat, November 8, **2002**.

[S23] OZM, http://www.ozm.cz. (June **2025**).

[S24] Impact: Insensitive >40 J, less sensitive ≤35 J, sensitive ≤4 J, very sensitive ≤3 J; friction: Insensitive >360 N, less sensitive=360 N, sensitive 80 N, very sensitive ≤80 N, extreme sensitive ≤10 N; According to the UN Recommendations on the Transport of Dangerous Goods (+) indicates: not safe for transport.

[S25] I. L. Dalinger, I. A. Vatsadze, T. K. Shkineva, G. P. Popova, S. A. Shevelev, *Synthesis* **2012**, *44*, 2058–2064.

[S26] I. L. Dalinger, I. A. Vatsadze, T. K. Shkineva, G. P. Popova, S. A. Shevelev, Y. V. Nelyubina, *J. Heterocycl. Chem.* **2013**, *50*, 911–924.
